# Phylogenomics and antimicrobial resistance of *Salmonella* Typhi and Paratyphi A, B and C in England, 2016–2019

**DOI:** 10.1099/mgen.0.000633

**Published:** 2021-08-09

**Authors:** Marie Anne Chattaway, Amy Gentle, Satheesh Nair, Laura Tingley, Martin Day, Iman Mohamed, Claire Jenkins, Gauri Godbole

**Affiliations:** ^1^​ Gastrointestinal Bacteria Reference Unit, National Infection Service, Public Health England, 61 Colindale Avenue, London NW9 5EQ, UK; ^2^​ Travel Health and IHR, National Infection Service, Public Health England, 61 Colindale Avenue, London NW9 5EQ, UK

**Keywords:** antimicrobial resistance, enteric fever, genomic, Paratyphi, phylogeny, Typhi

## Abstract

The emergence of antimicrobial resistance (AMR) to first- and second-line treatment regimens of enteric fever is a global public-health problem, and routine genomic surveillance to inform clinical and public-health management guidance is essential. Here, we present the prospective analysis of genomic data to monitor trends in incidence, AMR and travel, and assess hierarchical clustering (HierCC) methodology of 1742 isolates of typhoidal salmonellae. Trend analysis of *Salmonella* Typhi and *S*. Paratyphi A cases per year increased 48 and 17.3%, respectively, between 2016 and 2019 in England, mainly associated with travel to South Asia. *S*. Paratyphi B cases have remained stable and are mainly associated with travel to the Middle East and South America. There has been an increase in the number of *S*. Typhi exhibiting a multidrug-resistant (MDR) profile and the emergence of extensively drug resistant (XDR) profiles. HierCC was a robust method to categorize clonal groups into clades and clusters associated with travel and AMR profiles. The majority of cases that had XDR *S*. Typhi reported recent travel to Pakistan (94 %) and belonged to a subpopulation of the 4.3.1 (H58) clone (HC5_1452). The phenotypic and genotypic AMR results showed high concordance for *S*. Typhi and *S*. Paratyphi A, B and C, with 99.99 % concordance and only three (0.01 %) discordant results out of a possible 23 178 isolate/antibiotic combinations. Genomic surveillance of enteric fever has shown the recent emergence and increase of MDR and XDR *S*. Typhi strains, resulting in a review of clinical guidelines to improve management of imported infections.

## Data Summary

fastq sequences were deposited in the National Center for Biotechnology Information (NCBI) Sequence Read Archive under the BioProject accession number PRJNA248792 (www.ncbi.nlm.nih.gov/bioproject/?term=248792). Refer to Table S1 (available with the online version of this article) for SRA accession numbers.

**Impact Statement**

In this article, we present a trend analysis of *Salmonella enterica* subsp *. enterica* Typhi and Paratyphi A, B and C in England, from 2016 to 2019, with the use of genomics validated by phenotypic antimicrobial-sensitivity testing. This research shows the genetic complexity behind the phenotypic antimicrobial resistance (AMR) of typhoidal *Salmonella* and is the first study, to our knowledge, to analyse core-genome multilocus sequence typing (cgMLST) as a methodology to assess the phylogeny and understand transmission of resistance of all typhoidal *Salmonella* in England. This study shows an alarming increase of *S*. Typhi in England that can be traced back to an outbreak in Pakistan l. These surveillance data inform clinical management of cases, as antimicrobial therapy can be tailored to the individual based on their travel history, and public health guidance and advice for travelers to high risk destinations. These data are publicly available and can be used to better understand transmission and spread of AMR on a global scale.

## Introduction

Enteric fever, the collective term for typhoid and paratyphoid fevers, is caused by *Salmonella enterica* subspecies *enterica* serovar Typhi (*S*. Typhi) and Paratyphi A, B or C (*S*. Paratyphi), which are human-host-restricted pathogens causing systemic infection transmitted via contaminated food, water or contact with an infected case [1]. Although, globally, the number of cases declined by 44.8 % (25.9 million to 14.3 million cases) from 1990 to 2017, deaths from enteric fever in 2017 were estimated to be 135 900 with higher case fatality among children and older adults [2]. Fatalities and symptom severity rates can be reduced with prompt, appropriate treatment. However, over the last two decades, multiple-drug resistance [traditionally defined as resistance to amoxicillin (or ampicillin), co-trimoxazole and chloramphenicol] [3] and decreased susceptibility to ciprofloxacin have been described [4]. More recently, extended-spectrum β-lactamase (ESBL) producing strains of *S*. Typhi [5] and *S*. Paratyphi A [6] have emerged. Consequently, options of first-line antibiotics are limited, and clinical management of typhoid fever is becoming increasingly challenging. Recent outbreaks of extensively drug resistant (XDR) (resistance to three first-line drugs ampicillin, chloramphenicol and trimethoprim/sulphamethoxazole, as well as ciprofloxacin and third-generation cephalosporins) [7] *S*. Typhi have been described, limiting the treatment options even further [8].

Surveillance of antimicrobial resistance (AMR) in typhoidal salmonellae is essential to inform effective clinical management. Although enteric fever is associated with low-middle income countries (LMIC) where surveillance is limited, the majority of cases in the UK are travel related, with patients reporting travel to India, Pakistan and Bangladesh [9]. These data can be used as an informal sentinel AMR surveillance approach to assess emerging trends of resistance [10].

In 2014, Public Health England (PHE) started to routinely perform whole-genome sequencing (WGS) on all *Salmonella* isolates referred to the reference laboratory, transforming surveillance and facilitating real-time monitoring of genotypic AMR determinants [11]. Validation of sequenced-derived AMR determinants to infer phenotypic surveillance was undertaken with *S*. Typhi and *S*. Paratyphi A and B isolates submitted to PHE between April 2014 and August 2016, and showed 99.97 % concordance, leading to the conclusion that sequence data provided a robust and informative approach for monitoring multidrug resistance and emerging resistance in enteric fever strains [9]. However, understanding the population structure and the clonality of increases of cases are not prospectively integrated into routine surveillance. Despite the advance of genomic methodologies, public-health organizations still need to study approaches in developing, validating and analysing the vast amount of genomic data and integrate it into routine surveillance. A genotyping scheme for *S*. Typhi is available on GitHub [12] and has been shown to be useful for global sentinel surveillance [10]. However, it is not a readily available platform, requires specialized bioinformatic skills to run the program and is specific to *S*. Typhi. Hierarchical clustering (HierCC) of core-genome multilocus sequence typing (cgMLST) is a readily available platform on EnteroBase (https://enterobase.warwick.ac.uk/) and cgMLST sequence types (cgSTs) allow mapping of bacterial strains to a predefined population structure at multiple levels of resolution [13]. The aim of this study was to review the genomic data used for the surveillance of enteric fever in England to assess: (i) trends in the number of cases over the past 4 years; (ii) AMR trends; (iii) the emergence of any new AMR profiles, genes or clones; (iv) assessment of HierCC as a potential method for sentinel surveillance with respect to AMR profile and geographical origin of each isolate.

## Methods

### Bacterial strains

All isolates of *S*. Typhi and *S*. Paratyphi A, B and C referred to the Gastrointestinal Bacteria Reference Unit (GBRU), from local diagnostic laboratories in England, between first January 2016 and 31st December 2019 were included for this analysis. The invasive index can give an indication of how invasive a group of pathogens are by assessing how frequently they are isolated from blood sources versus other isolated sites, such as faeces [14]. The invasive index was calculated for *S*. Typhi, *S*. Paratyphi A and *S*. Paratyphi B, and was a ratio of isolates recovered from blood to the total number of isolates recovered (total of blood and faeces isolates, other sources or unknown sources were excluded) for each serovar.

### Epidemiology

Patient information, including demographics, symptoms, treatment and outcomes, was obtained by PHE using an enhanced surveillance questionnaire (www.gov.uk/government/publications/typhoid-and-paratyphoid-enhanced-surveillance-questionnaire). This also included questions pertaining to all destinations during any foreign travel that occurred during the likely incubation period (28 days before the onset of symptoms). No specific consent was required from the patients whose data were used in this analysis as PHE has authority to handle patient data for public-health monitoring and infection control under section 251 of the UK National Health Service Act of 2006. The sample date of duplicate isolates (more than one isolate from the same patient) was assessed, and *Salmonella* carriage (whether invasive from the blood or shed in the stool) was split into four categories: same episode (≤3 weeks from the first submitted isolate), convalescent carriage (>3 weeks – ≤3 months), temporary carriage (>3 months – 12 months) and chronic carriage (>12 months) [15, 16].

### WGS

Following DNA extraction at containment level 3, all 1742 isolates were prepared for sequencing with Nextera XT DNA preparation kits, and sequenced on the Illumina HiSeq 2500 platform in rapid run mode to produce 100 bp paired-end reads. Trimmomatic v0.40 [17] was used to quality trim fastq reads with bases removed from the trailing end that fell below a PHRED score of 30. The Metric Orientated Sequence Type (most) v1 [18] was used for sequence type (ST) assignment and identification assigned using the Salmonella MLST database [19].

AMR determinants were sought using Genefinder v1–5, as previously described [9]. Known acquired resistance genes and resistance-conferring mutations relevant to β-lactams (including carbapenems), fluoroquinolones, aminoglycosides, chloramphenicol, macrolides, sulphonamides, tetracyclines, trimethoprim, rifamycins and fosfomycin, and acquired genes associated with colistin resistance, were included in the analysis [9, 20]. β-Lactamase variants were determined with 100 % identity using the reference sequences downloaded from the Lahey (www.lahey.org) or National Center for Biotechnology Information (NCBI) (www.ncbi.nlm.nih.gov/pathogens/beta-lactamase-data-resources) β-lactamase data resources. Reference sequences for acquired resistance genes were curated from those described in the Comprehensive Antimicrobial Resistance Database (http://arpcard.mcmaster.ca) and the ResFinder datasets (https://cge.cbs.dtu.dk/services/data.php). Chromosomal mutations were based on previously published variations in the quinolone-resistance-determining regions (QRDRs) of *gyrA, gyrB, parC* and *parE*, which are associated with resistance to quinolones. ST, eBURST group (eBG) and serotype were determined from the genome data [18, 19].

### Antimicrobial-susceptibility testing

Three *S*. Typhi and one *S*. Paratyphi A isolates were not phenotypically tested as they were non-viable at the time of testing, leaving a total of 1738 isolates available for phenotypic testing. Susceptibility testing was performed retrospectively on all isolates recovered from the PHE archive based on the EU (European Union) protocol for the monitoring of AMR [21]. Minimum inhibitory concentrations (MICs) were determined in containment level 3 by agar dilution using Mueller–Hinton agar for the following antimicrobials: amoxicillin, amoxicillin/clavulanic acid (*S*. Typhi only), ceftriaxone, ceftazidime, ertapenem, ciprofloxacin, gentamicin, azithromycin, tetracycline, fosfomycin, trimethoprim, colistin, chloramphenicol and trimethoprim/sulphamethoxazole. Breakpoints and screening concentration criteria used for interpretation were as recommended by EUCAST (European Committee on Antimicrobial Susceptibility Testing) [22]. Rifampicin susceptibility was performed only where there were genomic resistance markers to rifamycins as this was not previously assessed. Streptomycin was not tested phenotypically as it was previously validated against genomic markers [9] and has since been removed as a recommended antibiotic to screen for surveillance [21]

### Population structure of *S*. Typhi

Raw sequence data files of isolates from cases based in England were uploaded to EnteroBase (https://enterobase.warwick.ac.uk/) and short reads were assembled by EnteroBase using the then current backend pipelines (versions 3.61–4.1) including cgMLST analysis to produce a cgST as previously described [23] using the cgMLST v2 HierCC v1 algorithm [24]. There were 1455 isolates that met the cgMLST quality parameters for *Salmonella* (minimum size 4000 kbp, maximum size 5800 kbp, minimum N50 20 kbp, maximum number contigs 600, maximum low-quality sites 5 %, minimum taxonomic purity 70 % [13]) and 860 cases of *S*. Typhi, 529 cases of *S*. Paratyphi A and 65 cases of *S*. Paratyphi B were included for analysis. *S*. Paratyphi C was not further analysed since it was a single isolate. The minimum spanning tree was created in EnteroBase for each pathogen using the MSTree v2 algorithm and visualizing on GrapeTree [24]. Previous studies have shown that analysing strains at the 5 SNP threshold might be appropriate to detect clusters or closely related clones, and that cgMLST is equivalent to SNP when detecting clusters [23, 25–27]. Therefore, HierCC was analysed at the five allelic level (HC5 – strains linked within five cgMLST alleles) for trend analysis in association with travel and resistance patterns. Phylogenetic analysis was undertaken using the Ninja Neighbour Joining method [28] and visualized on iTOL v5 [29].

### Data access

fastq sequences were deposited in the National Center for Biotechnology Information Sequence Read Archive (SRA) under BioProject accession number PRJNA315192 and the SRA numbers are available in Table S1.

## Results

### Demographic data

**Fig. 1.**
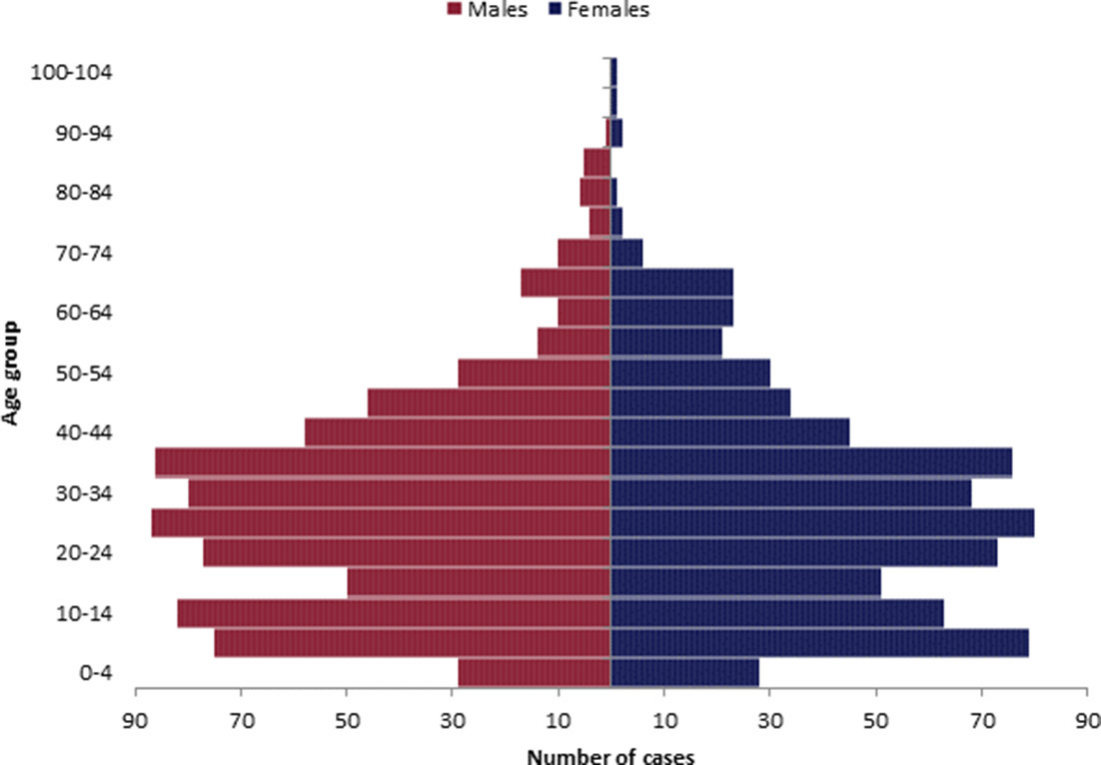
Population pyramid of age and sex distribution of 1473 cases of enteric fever, from 2016–2019.

A total of 1742 isolates of *S*. Typhi (*n*=1037), *S*. Paratyphi A (*n*=608), *S*. Paratyphi B (*n*=96) and *S*. Paratyphi C (*n*=1) were received by the Gastrointestinal Bacteria Reference Unit (GBRU), and identification was confirmed using WGS. There were 1742 isolates from 1473 patients, with 236 patients having additional isolates referred after the initial isolation. Patients reported to general practitioners or local hospitals in England with diarrhoea, abdominal pain and/or symptoms consistent with enteric fever (*S*. Typhi patients *n*=870, *S*. Paratyphi A patients *n*=535, *S*. Paratyphi B patients *n*=67, *S*. Paratyphi C patients *n*=1 in 2019). From 2016 to 2019, the number of cases of *S*. Typhi increased from 165 to 317 (2016, *n*=165; 2017, *n*=186; 2018, *n*=202; 2019, *n*=317). The number of cases of *S*. Paratyphi A was lowest in 2017 (*n*=102) and highest in 2019 (*n*=170). The number of cases of *S*. Paratyphi B remained stable (*n*=15, *n*=15, *n*=18, *n*=19, respectively) and there was only one case of *S*. Paratyphi C, in 2019. Of the 1473 cases, 766 of the patients were male (52 %) and 707 were female (48 %), with the most common age range for infection being between 5–14 years and 20–39 years old (Fig. 1). The age and sex distribution were similar when analysed by individual serovar (data not shown).

**Fig. 2.**
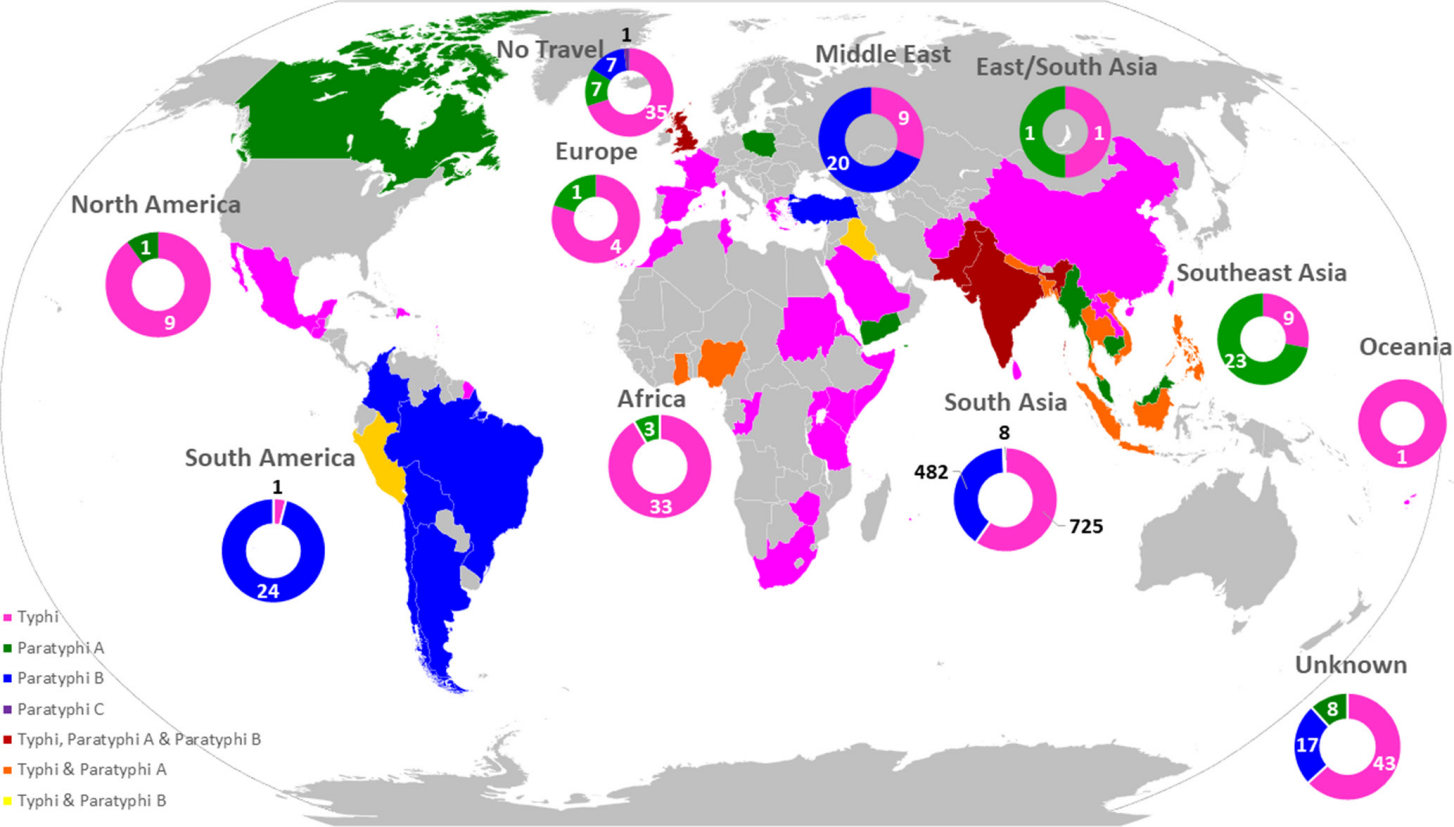
Travel of patients within 28 days of generating symptoms.

**Fig. 3.**
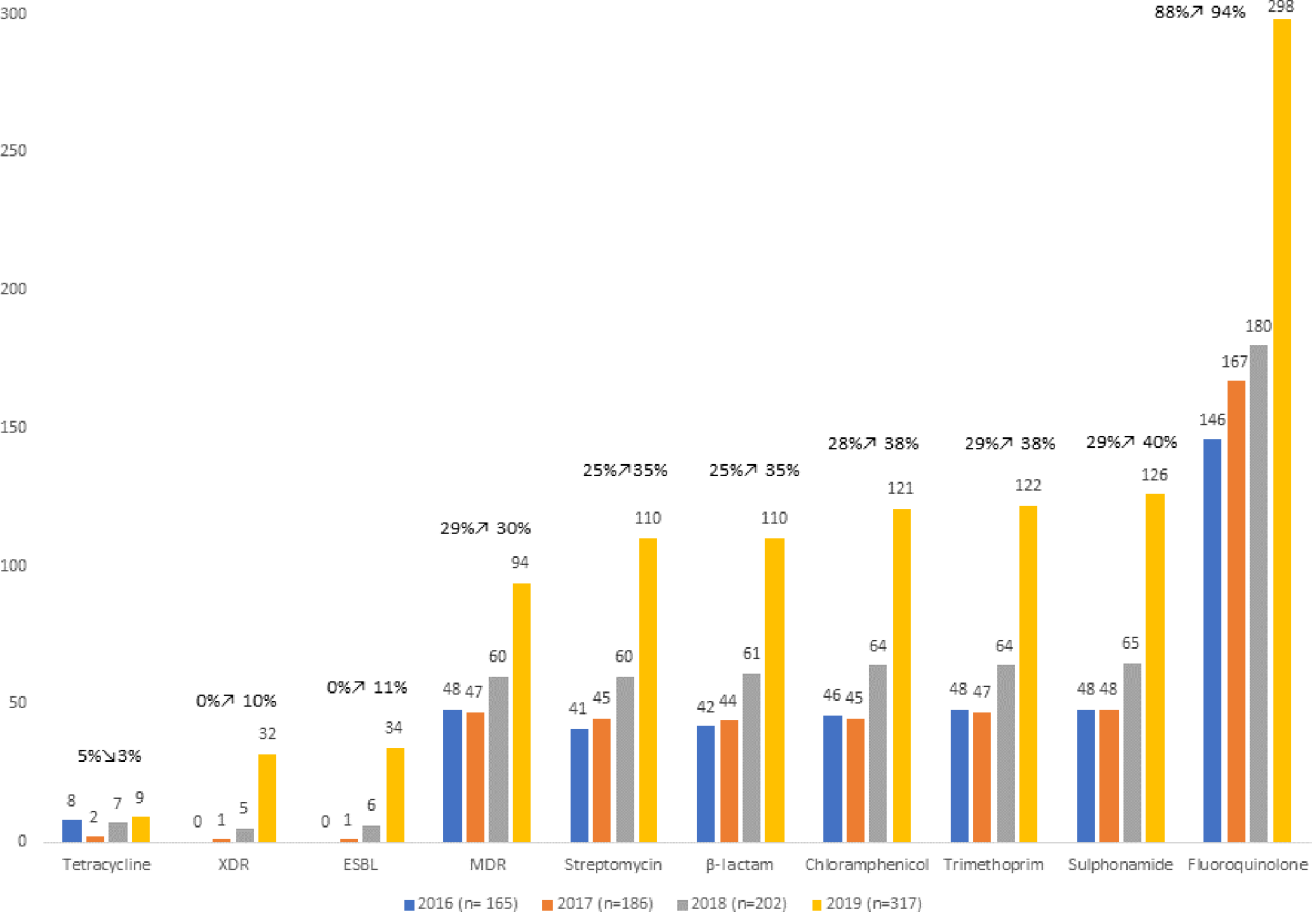
Antibiotic-resistance trends in *S*. Typhi, 2016–2019. Antibiotic-resistance trends of *S*. Typhi cases received between 2016 and 2019 based on the presence of AMR determinant markers. Categories are subdivided into antibiotic classes and strains are classified as β-lactams (Amoxicillin), extended β-lactamase producers (ESBL), multidrug resistant (MDR) and extremely drug resistant (XDR). With the exception of tetracycline, *S*. Typhi strains have increased resistance to all classes of antibiotics, including the increase of ESBL, MDR and XDR strains.

Of the 1473 cases presenting with enteric fever, 1366 (92.7 %) patients reported a history of foreign travel within a 28 day period prior to the onset of symptoms (54.3 % *S*. Typhi; 34.8 % *S*. Paratyphi A; 3.6 % *S*. Paratyphi B). There were 50 (3.4 %) cases for which no foreign travel was reported and travel information was unknown for 57 (3.9 %) patients. The most frequently visited countries varied depending on serovar, with *S*. Typhi and *S*. Paratyphi A cases associated with travel to South Asia, including India [*S*. Typhi, *n*=320/801 (40.0 %); *S*. Paratyphi A, *n*=258/513 (50.3 %)], Pakistan [*S*. Typhi, *n*=344/801 (42.9 %); *S*. Paratyphi A, *n*=173/513 (33.7 %)] and Bangladesh [*S*. Typhi, *n*=55/801 (6.9 %); *S*. Paratyphi A, *n*=49/513 (9.6 %)] (Fig. 2). Whereas *S*. Paratyphi B was mostly associated with travel to Bolivia/Peru, South America [*S*. Paratyphi B, *n*=21/52 (40.4 %)] or Iraq/the Middle East [*S*. Paratyphi B, *n*=17/52 (32.7 %)] (Fig. 2). The highest increase of travel-associated cases from a single country was *S*. Typhi cases from Pakistan, which increased from 60/144 (41.7 %) cases in 2016 to 174/300 (58 %) cases in 2019. Travel from Pakistan to England, in both UK and overseas residents, had steadily increased between 2016 and 2019 (2016, *n*=697 933; 2017, *n*=830 611; 2018, *n*=828 682; 2019, *n*=858 089; Table S1).The most notable increase of enteric fever cases was with *S*. Typhi in which there has been a steady increase of approximately 20 extra *S*. Typhi cases (increase of 10 %) in England per year since 2016, with the most notable increase of cases from 2018 (*n*=202) to 2019 (*n*=317) (increase of 36.3 %) (Fig. 3). This large increase can be explained with the increase in reported travel of *S*. Typhi patients to Pakistan, which rose by 63 % (from 68 cases to 184 cases per year). Positive cases of *S*. Typhi and *S*. Paratyphi A in the population from returning travellers from Pakistan doubled (from 0.01 to 0.02% and 0.005 to 0.01%, respectively) in 2019 (Table S1).

### Microbiology

Of the 1742 isolates received by PHE, 992 (56.9 %) were cultured from blood (*S*. Typhi, *n*=597, 34.3 %; *S*. Paratyphi A, *n*=362, 20.8 %; *S*. Paratyphi B, *n*=32, 1.8 %; *S*. Paratyphi C, *n*=1, 0.05 %), 451 (25.9 %) were from faeces (*S*. Typhi, *n*=263; *S*. Paratyphi A, *n*=138; *S*. Paratyphi B, *n*=50) and 33 (1.9 %) were derived from various specimens including abscesses and urine samples, and the infection site was not stated for the remaining 266 (15.3 %) (*S*. Typhi, *n*=159; *S*. Paratyphi A, *n*=95; *S*. Paratyphi B, *n*=12). The invasive index (based on blood and faeces sources received) was highest for *S*. Paratyphi A (72.4) and *S*. Typhi (69.4) and the lowest with *S*. Paratyphi B (39.0) [14].

The isolates of *S*. Typhi belonged to eBG13 (ST1, *n*=771; ST2, *n*=245; ST2173, *n*=5; ST2209, *n*=10; ST4760, *n*=3; ST5883, *n*=1; ST6142, *n*=2). *S*. Paratyphi A isolates were found in eBG11 (ST85, *n*=341; ST129, *n*=257; ST1938, *n*=5; ST1939, *n*=2, ST7555, *n*=3). All *S*. Paratyphi B isolates belonged to eBG5 (ST86, *n*=96) and the single isolate of *S*. Paratyphi C belonged eBG20, ST146.

### Comparison between phenotypic and genotypic AMR

Concordance between phenotypic and genotypic AMR results was high, ranging from 99.9 to 100 % with the 14 antibiotics tested in *S*. Typhi (*n*=1034) and 13 antibiotics tested in *S*. Paratyphi A (*n*=607), *S*. Paratyphi B (*n*=96) and *S*. Paratyphi C (*n*=1). There were three (0.01%) discordant results out of a possible 23 178 isolate/antibiotic combinations (Tables 1, S1 and S2).

**Table 1.** Evaluation of genotypic analysis for the prediction of resistance phenotypes for *S.* Typhi Genotype resistance is classified as having one or more genetic marker/mutation.

Antibiotic	MIC no.*	Phenotype: susceptible		Phenotype: resistant	
		Genotype: resistant	Genotype: susceptible	Genotype: resistant	Genotype: susceptible
AMX	1034	0	726	308	0
AMX-CL	1034	0	726	308	0
CAZ	1034	0	983	51	0
CRO	1034	0	983	51	0
ETP	1034	0	1034	0	0
GEN	1034	0	1034	0	0
CIP	1034	0	83	950	**1**
AZM	1034	0	1034	0	0
TMP	1034	**1**	704	328	**1**
FOS	1013	0	1012	1	0
TET	1034	0	1006	28	0
SXT	1034	0	698	336	0
CHL	1034	0	711	323	0
COL	1034	0	1034	0	0
Total combinations	14 455	–	–	–	–

AMX, Amoxicillin; AMX-CL, amoxicillin/clavulanic acid; CAZ, ceftazidime; CIP, ciprofloxacin; CRO, ceftriaxone; ETP, ertapenem; GEN, gentamicin; AZM, azithromycin; TMP, trimethoprim; FOS, fosfomycin; TET, tetracycline: SXT, trimethoprim/sulphonamide; CHL, chloramphenicol; COL, colistin. Numbers in bold relate to descrepancies between genotype and phenotype.

*The number of isolates that had phenotypic MIC testing.

Discordance between a phenotypically sensitive isolate and a genotypically resistant profile was associated with trimethoprim (*n*=1/1034, 0.1 %, *S*. Typhi isolate 229 163, MIC=<0.5 mg l^−1^) where the isolate was positive for the *dfrA-7* gene. Discordance between a phenotypically resistant isolate and a genotypically sensitive profile (no genetic markers) was found in one isolate associated with ciprofloxacin (*n*=1/1034, 0.1%, *S*. Typhi isolate 474 628, MIC=0.125 mg l^−1^) and one isolate associated with rifampicin (*n*=1/1034, 0.1 %, *S*. Typhi isolate 356 314, MIC=>32 mg l^−1^) (Tables S1 and S2).

There were three genes/mutations found that did not confer phenotypic resistance: single mutation *gyrB*[465:Q-L] (*n*=3/1034, 0.3 %, *S*. Typhi isolates 790 549, 773 645 and 789 981, MIC=<0.015 mg l^−1^ ciprofloxacin); single mutation *parC*[57:T-S] (*n*=16/607, 2.6 %, *S*. Paratyphi A, MIC=<0.06 mg l^−1^ ciprofloxacin) and presence of the *tet*(Q) gene (*n*=1/1034 *S*. Typhi isolate, 579 104, MIC=<2 mg l^−1^ tetracycline). The *gyrB*[465:Q-L] mutation and *tet*(Q) gene were not previously detected by Day *et al.* (in 2018), who assessed genotypic resistance markers in *S*. Typhi between 2014 and 2016 [9]. This study confirmed previous findings that the *parC*[57:T-S] mutation was identified in all isolates of *S*. Paratyphi A [9] (Table S1) and provides further evidence that, when present with no additional mutations in the DNA gyrase or topoisomerase genes, this mutation does not confer reduced susceptibility [30].

Genes [*aac(6′)-Iaa* – aminoglycoside acetyltransferase, and *aph(6)-Id* – aminoglycoside phosphotransferase] predicting for the modification of aminoglycoside enzymes and potentially conferring resistance to amikacin and tobramycin [31, 32] were detected; however, phenotype testing of amikacin and tobramycin are not routinely carried out at PHE. The *aac(6′)-Iy* gene was also detected but is intrinsic and does not confer resistance to aminoglycoside in enteric *Salmonella* unless additional factors, such as a transcriptional fusion, have occurred [33].

### Chronic carriage and resistance

There were 232 patients where additional isolates were received after the initial isolation and were classified as follows: 183 patient isolates were classified as being from the same episode, 38 patient isolates were classified as being from a convalescent carrier, 8 patient isolates were classified as being from a temporary carrier, and 3 patient isolates were classified as being from a chronic carrier. Isolates from carriers were not associated with an increased resistance profile and carriage was associated with the gut and persistent invasive disease (Table 2).

**Table 2.** Carriage and AMR status of typhoidal *Salmonella
* Table showing the AMR patterns and carriage status in patients with multiple isolate referrals. Percentage is in relation to the grand total of each antibiotic resistance pattern in each carriage cateogory by pathogen. Numbers in brackets are isolates from blood.

Organism and resistance	Carriage status								
	Same episode (≤3 weeks)		Convalescent carrier (3 weeks–≤3 months)		Temporary carrier (3–≤12 months)		Chronic carrier (>12 months)		Grand total
	**No.**	**%**	**No.**	**%**	**No.**	**%**	**No.**	**%**	**No.**
*** Salmonella* Typhi**	**115** (60)	**79.3** (41.4)	**24** (15)	**16.6** (10.3)	**4** (2)	**2.8** (1.4)	**2** (1)	**1.4**	**145** (78)
Ciprofloxacin resistant	68 (41)	46.9	15 (8)	10.3	2 (2)	1.4	1	0.7	86 (51)
Ciprofloxacin/rifamycin resistant	0	0.0	0	0.0	1	0.7	0	0.0	1 (0)
Fully susceptible	7 (2)	4.8	3 (3)	2.1	1	0.7	0	0.0	11 (5)
MDR	33 (16)	22.8	5 (3)	3.4	0	0.0	1 (1)	0.7	39 (20)
XDR	7 (1)	4.8	1 (1)	0.7	0	0.0	0	0.0	8 (2)
*** Salmonella* Paratyphi A**	**56** (35)	**82.4** (51.5)	**10** (7)	**14.7** (10.3)	**2**	**2.9**	**0**	**0.0**	**68** (42)
Ciprofloxacin resistant	54 (33)	79.4	9 (6)	13.2	2	2.9	0	0.0	65 (39)
Fully susceptible	2 (2)	2.9	1 (1)	1.5	0	0.0	0	0.0	3 (3)
*** Salmonella* Paratyphi B**	**12** (11)	**63.1** (57.9)	**4** (2)	**21.1** (**10.5**)	**2**	**10.5**	**1**	**5.3**	**19** (13)
Ciprofloxacin resistant	1 (1)	5.3	1	5.3	0	0.0	0	0.0	2 (1)
Fully susceptible	11 (10)	57.9	3 (2)	15.8	2	10.5	1	5.3	17 (12)
**Grand Total**	**183** (106)	**78.9** (45.7)	**38** (24)	**16.4** (10.3)	**8** (2)	**3.4** (0.9)	**3** (1)	**1.3** (0.4)	**232** (133)

### Genomic trends of resistance in *S*. Typhi

HierCC and resistance trend analysis of *S*. Typhi. *S*. Paratyphi A and *S*. Paratyphi B showed clonal groups associated with travel and resistance, particularly with an increase of resistance in *S*. Typhi in the majority of antibiotic classes (Fig. 3, Table S3). Since the previous study describing AMR from April 2014 to August 2016 [9], there has been an increase in AMR in *S*. Typhi to most classes of antibiotics, in addition to the detection of new mutations/combinations of genes conferring resistance (Table 3, Fig. 3). The most common resistance for *S*. Typhi was to ciprofloxacin (*n*=509/970, 52.5 %). The largest increase of resistance was resistance to sulphonamides, which was previously reported as 22.9 % in 2015 [9] and has risen to 40 % in 2019 (Fig. 3). The highest clinical impact of change in genomic resistance is the recent introduction of ESBL and XDR types in *S*. Typhi, which accounted for 10.7 and 10.1 % of isolates in 2019, respectively (Table 3, Fig. 3). Based on the WGS prediction, 803/870 (92.3 %) isolates from cases of *S*. Typhi were resistant to at least one antimicrobial agent, compared with *S*. Paratyphi A (*n*=523/535, 97.8 %), *S*. Paratyphi B (*n*=8/67, 11.9 %) and *S*. Paratyphi C (*n*=0/1, 0 %). Further details are described below.

**Table 3.** Overview of genomic resistance of typhoidal *Salmonella
* Table describing the number of isolates (one representative per case) with genomic AMR for *S.* Typhi and *S.* Paratyphi A, B and C. Per cent values in bold show an increase of resistance from 2016 and 2019 or since the last reported study [9].

Antibiotic class or resistance type	2016		2017		2018		2019		Grand total		Previous study %*
	No.	%	No.	%	No.	%	No.	%	No.	%	
***S*** **. Typhi – no. of cases**	165	−	186	−	202	−	317	−	870	−	−
Tetracycline	8	4.8	2	1.1	7	3.5	9	2.8	26	**3.0**	1.8
XDR	0	0.0	1	0.5	5	2.5	32	**10.1**	38	**4.4**	0
ESBL producer	0	0.0	1	0.5	6	3.0	34	**10.7**	41	**4.7**	0
β-Lactam	42	25.5	44	23.7	61	30.2	110	**34.7**	257	**29.5**	23.2
Streptomycin	41	24.8	45	24.2	60	29.7	110	**34.7**	256	**29.4**	22.9
Chloramphenicol	46	27.9	45	24.2	64	31.7	121	**38.2**	276	**31.7**	23.8
Trimethoprim	48	29.1	47	25.3	64	31.7	122	**38.5**	281	**32.3**	24.7
MDR	48	29.1	47	25.3	60	29.7	94	**29.7**	249	**28.6**	13.8
Sulphonamide	48	29.1	48	25.8	65	32.2	126	**39.7**	287	**33.0**	25
Fluoroquinolone#	146	88.5	167	89.8	180	89.1	298	**94.0**	791	**90.9**	84.3
Fosfomycin	0	0.0	0	0.0	1	0.5	0	0.0	1	**0.1**	0
Rifamycin	0	0.0	1	0.5	0	0.0	0	0.0	0	0.0	0
Colistin	0	0.0	0	0.0	0	0.0	0	0.0	0	0.0	0
Tetracycline	8	4.8	2	1.1	7	3.5	9	2.8	26	**3.0**	1.8
***S.*** **Paratyphi A – no. of cases**	**142**	–	**102**	–	**121**	–	**170**	–	**535**	–	–
β-Lactam/ESBL producer	0	0.0	1	1.0	0	0.0	0	0.0	1	**0.2**	0.0
Fluoroquinolone#	135	95.1	101	99.0	118	97.5	168	98.8	522	**97.6**	92.1
Rifamycin	1	0.6	0	0.0	0	0.0	0	0.0	1	**0.2**	0.0
***S.*** **Paratyphi B – no. of cases**	**15**	–	**15**	–	**18**	–	**19**	–	**67**	–	–
Fluoroquinolone#	2	13.3	1	6.7	3	16.7	2	10.5	8	11.9	13.6
***S*. Paratyphi C – no. of cases**	**0**	–	**0**	–	**0**	–	**1**	–	**1**	–	–
Any of the above tested antibiotics	0	0.0	0	0.0	0	0.0	0	0.0	0	0.0	n/a

*Mean per cent of genomic resistance determinants from isolates of enteric fever reported between April 2014 and August 2016 [9].

#Genetic Fluoroquinolone resistance varies between pathogens, for *S*. Typhi, resistance was classified as the presence of one marker/mutation, For *S*. Paratyphi A, B and C, resistance was classified as the presence of two or more genetic markers/mutations as the presence of a single marker such as the parC_ [57:T-S] mutation does not confer resistance.

N/A - not applicable

### Resistance to β-lactams, aminoglycosides, sulphonamides, trimethoprim, tetracyclines and phenicols and ESBL strains in *S.* Typhi

Of the 870 isolates of *S*. Typhi, 256 (29.4 %) had *bla*_TEM-1_, predicted to confer resistance to ampicillin. Determinants conferring resistance to β-lactam antibiotics, previously undetected in isolates of *S*. Typhi submitted to PHE, included *bla*_TEM-10_ (*n*=1), *bla*_CTX-M-15_ (*n*=39), *bla*_CTX-M-55_ (*n*=1) and *bla*_SHV-12_ (*n*=1). Genes predicted to confer resistance to streptomycin (*strA*, *strB*, *n*=256, 29.4 %) were detected. No phenotypic or genotypic resistance to gentamicin was detected and there were no 16S rRNA methyltransferase genes identified. There were 281 (32.3 %) isolates harbouring *dfrA* alleles (*dfrA1*, *n*=1; *dfrA7*, *n*=262; *dfrA14*, *n*=3; *dfrA1*5, *n*=7; combined *dfrA7*/*dfrA14*, *n*=8), conferring resistance to trimethoprim (Table S1). Sulphonamide resistance, encoded by *sul1* and/or *sul2* genes, was detected in 287 (33.0 %) isolates (Table S1). Twenty-five (2.9 %) isolates had *tetA* (Table S1) and no other tetracycline resistance determinants were detected. Chloramphenicol resistance, encoded by the *catA1* gene, was detected in 276 (31.7 %) isolates (Table S1).

### Resistance to quinolones in *S.* Typhi

Single mutations in the QRDR were previously defined as having reduced susceptibility [9, 10, 12]. However, due to fluoroquinolone treatment failure in patients with *S*. Typhi [22], isolates are clinically reported as resistant where the MIC is >0.06 mg l^−1^.

**Fig. 4.**
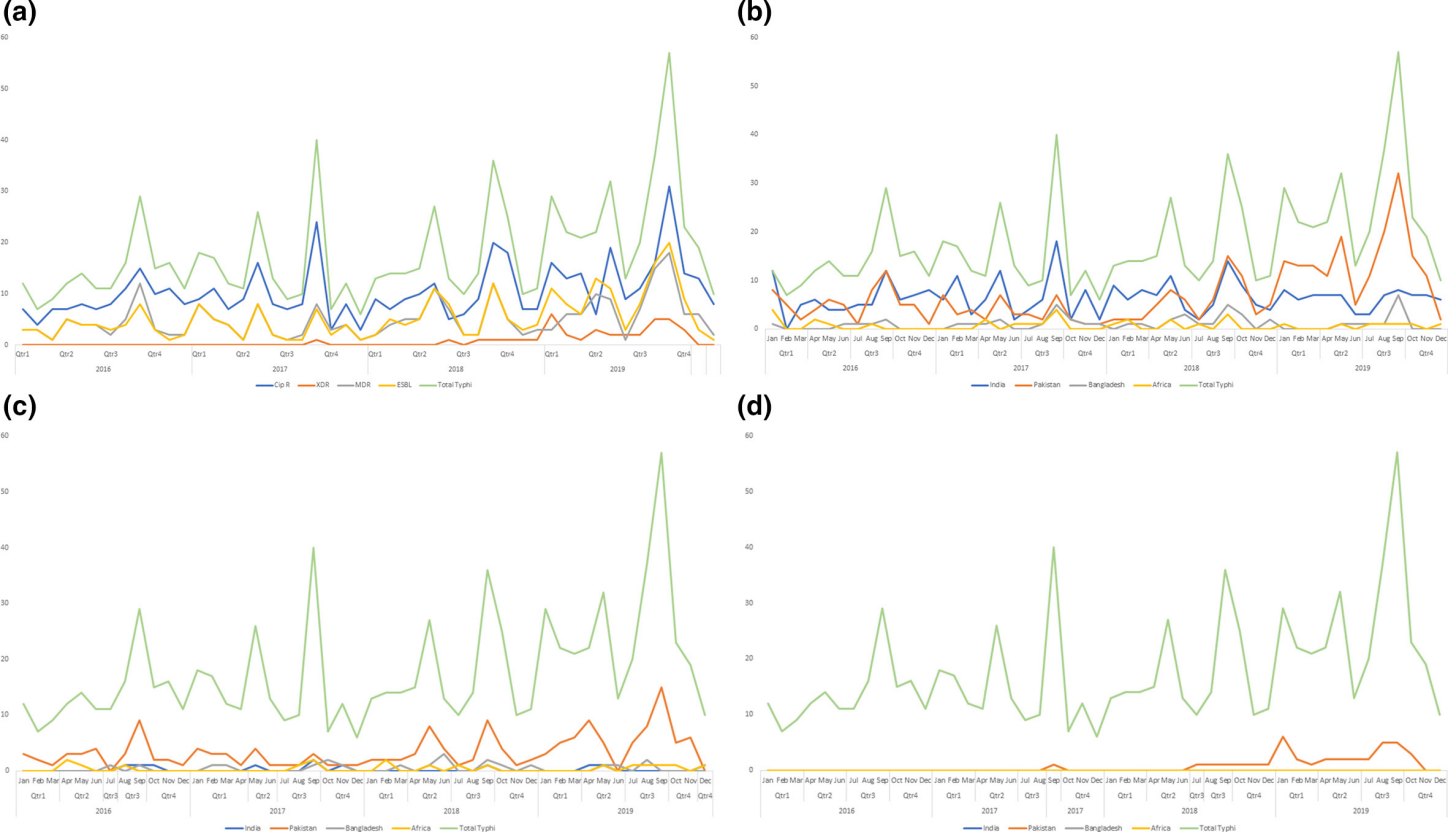
Trends of ciprofloxacin resistance in *S*. Typhi associated with travel to India, Pakistan, Bangladesh and Africa with y-axis showing the number of isolates. (a, b) Trends of resistance in *S*. Typhi cases received between 2016 and 2019 based on the presence of AMR determinant markers and subdivided into four main categories (Cip R, ciprofloxacin-resistant strains; ESBL, extended spectrum β-lactamase producing strains; MDR, multidrug resistant strains; XDR, extensively drug resistant strains) (a) and trends of ciprofloxacin resistance in *S*. Typhi associated with travel to India, Pakistan, Bangladesh and Africa (b). (c, d) Trends of MDR (c) and XDR (d) *S*. Typhi associated with travel to India, Pakistan, Bangladesh and Africa. Figures show an increase of *S*. Typhi Cip R, ESBL, MDR and XDR strains over time, with the largest increase in 2019 and with travel to Pakistan.

Of the 870 isolates from cases of *S*. Typhi in this study, 797 (91.6 %) exhibited either single mutations in *gyrA* (*n*=574) or *gyrB* (*n*=27), double mutations in *gyrA/parC* (*n*=24), *gyrA/parE* (*n*=19), *gyrA* (*n*=2) or *gyrA /gyrB* (*n*=2), triple mutations in *gyrA/parC* (*n*=101) or *gyrA/ParE* (*n*=3) (Table S1), potentially reducing ciprofloxacin treatment options. Cases infected with isolates of *S*. Typhi exhibiting resistance to ciprofloxacin resistance have increased over the years in *S*. Typhi, and were most commonly associated with travel to India and Pakistan (Fig. 4). The increase of *qnrS*-1 is linked with two clonal groups: the XDR *S*. Typhi strains in HC5_1452 associated with travel to Pakistan and the MDR *S*. Typhi strains in HC5_202 associated with travel to Zimbabwe (Tables S1 and S3, Fig. S1a–c).

### Resistance to macrolides, rifamycins, fosfomycin and colistin in *S.* Typhi

One isolate was predicted to be resistant to fosfomycin, encoded by the *fosA-v3* gene, and was phenotypically resistant [MIC >512 mg l^−1^]. None of the isolates were predicted to be resistant to the macrolides, rifamycin (though one isolate was phenotypically resistant) or colistin, and the *fosA* gene was not previously detected in the Day *et al.* (2018) study [9] (Tables 3 and S1).

### MDR and XDR *S*. Typhi

**Fig. 5.**
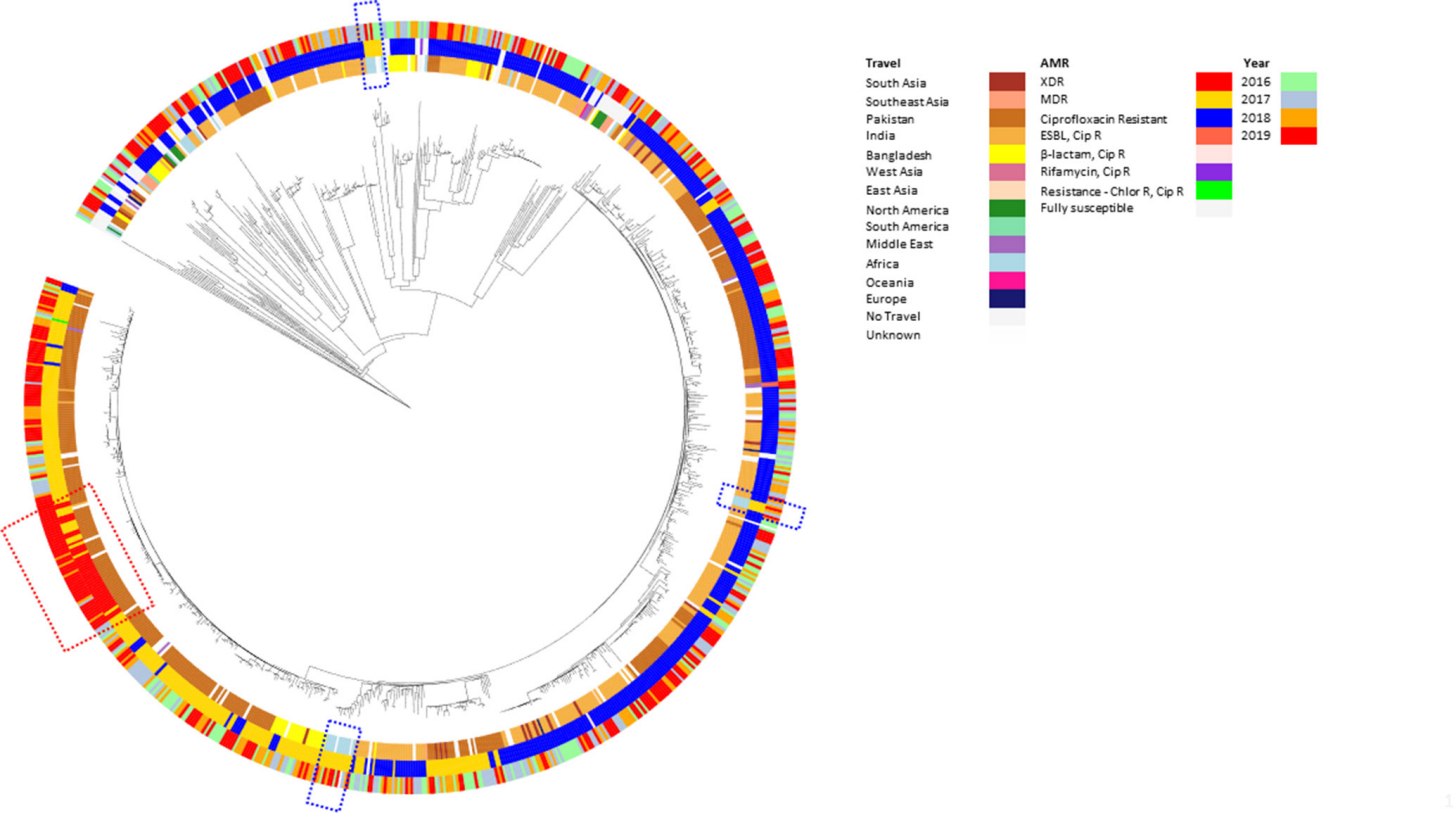
Neighbour-joining phylogeny of *S*. Typhi in association with year of receipt, AMR and travel. Phylogenetic tree of cgMLST analysis of *S*. Typhi strains generated using the neighbour-joining method and mapped against three categories: inner ring, travel; middle ring, AMR (XDR, extensively drug resistant strains; MDR, multidrug resistant strains; Cip R, ciprofloxacin-resistant strains; ESBL, extended-spectrum β-lactamase producing strains; Chlor R, chloramphenicol-resistant strains); outer ring, year. Multidrug resistance is predominantly associated with the HC5_1452 cluster associated with travel to Pakistan containing a sub-cluster of XDR strains (red dashed line box), predominantly occurring in 2019. Other smaller MDR clusters are distributed throughout the phylogeny and are associated with different regions of Africa (blue dashed line box).

The prevalence of MDR strains of *S*. Typhi has remained relative stable over the years with a slight increase since the last reported study in 2018 [9] (Table 3). The second most common genotypic resistance profile for *S*. Typhi was *TEM-1*;*strA*;*strB*;*gyrA*[83:S-F];*dfrA-7*;*sul-1*;*sul-2*;*catA-1* (*n*=177). Quinolone mutations other than those in *gyrA*, such as *qnrS* and *gyrB*, grouped into distinct HC5 clusters associated with certain travel to specific countries (Table S3). The increase of MDR strains was mainly associated with clusters from patients reporting travel to Pakistan [HC5_1452, HC5_7138, HC5_120934 (*n*=249/880, 28.3 %) (Figs 4, 5 and S1a–c, Table S3]. Other smaller MDR clusters were associated with patients reporting travel to Zimbabwe (HC5_202, *n*=11, 1.3 %) and Nigeria or Ghana (HC5_3475, HC5_49387, HC5_121018, HC5_220380, HC5_121042, *n*=7, 0.8 %) and have remained relatively consistent in numbers over the time frame of the study (Figs 4 and S1a–c, Table S3). Phylogenomics of *S*. Typhi in England confirmed the persistence of the dominating global MDR clone, also known as the global H58 clone or 4.3.1 clade [12], due to travel to South Asia as previously described [10] (Fig. 5). The most notable trend was the emergence of an XDR strain, which was first isolated from a traveller returning to the UK from Pakistan in 2017 [6] where isolations continued to increase throughout the study period. These XDR strains were found within a sub-cluster in HC5_1452 (*n*=37, 4.3 %) in association with travel to Pakistan (Figs 4, 5 and S1a–c, Table S3). The majority of the increase of *S*. Typhi has occurred in the last year (2018–2019) including MDR (HC5_1452, HC5_ 6578, HC_7138), XDR (HC5_1452) and ciprofloxacin (HC5_1452, HC5_2347, HC5_6578) strains belonging to clonal groups with travel from Pakistan and to a lesser extent India (Fig. S1a–c, Table S3). No other enteric fever pathogens other than *S*. Typhi were MDR or XDR (Tables 3 and S1).

### AMR of *S*. Paratyphi A, B and C

Of the 535 isolates from cases of *S*. Paratyphi A in this study, there was 1 isolate resistance to ampicillin and the third-generation cephalosporins encoded by two resistance determinants, *bla*_TEM-191_ and *bla*_CTX-M-15_ [6]. There were no other genes predicted to confer resistance to β-lactams, aminoglycosides sulphonamides, trimethoprim, tetracyclines or phenicols in *S.* Paratyphi A, B or C (Table S1).

**Fig. 6.**
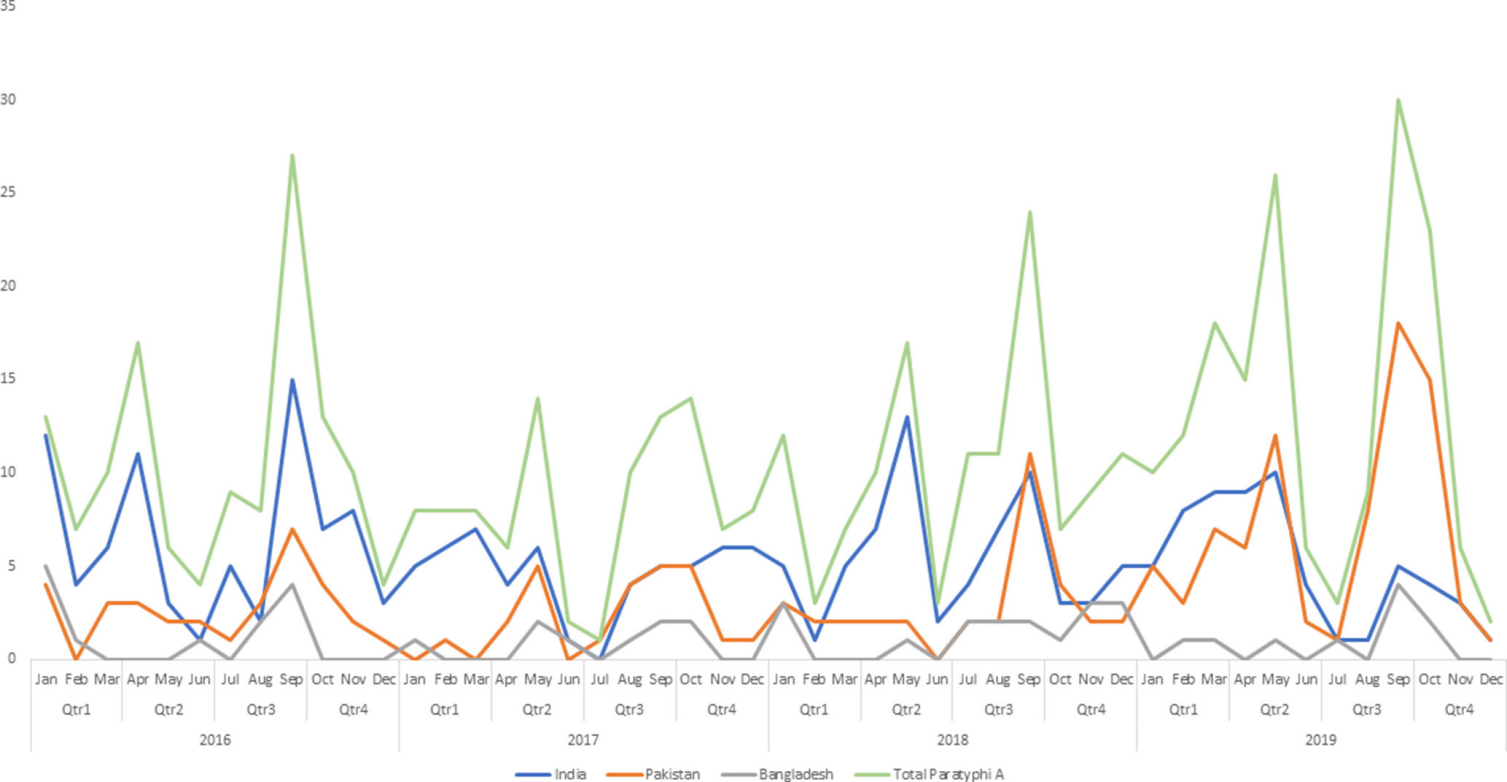
Trends of ciprofloxacin resistance in *S*. Paratyphi A associated with travel to India, Pakistan and Bangladesh with y-axis showing the number of isolates. Trends of ciprofloxacin-resistant *S*. Paratyphi A cases received between 2016 and 2019 based on the presence of ciprofloxacin AMR determinant markers. The figure shows seasonal trends with the seasonal peak in September/October and the trough in June/July. Resistance is mainly associated with travel to India until the last quarter of 2019, where travel to Pakistan increases.

**Fig. 7.**
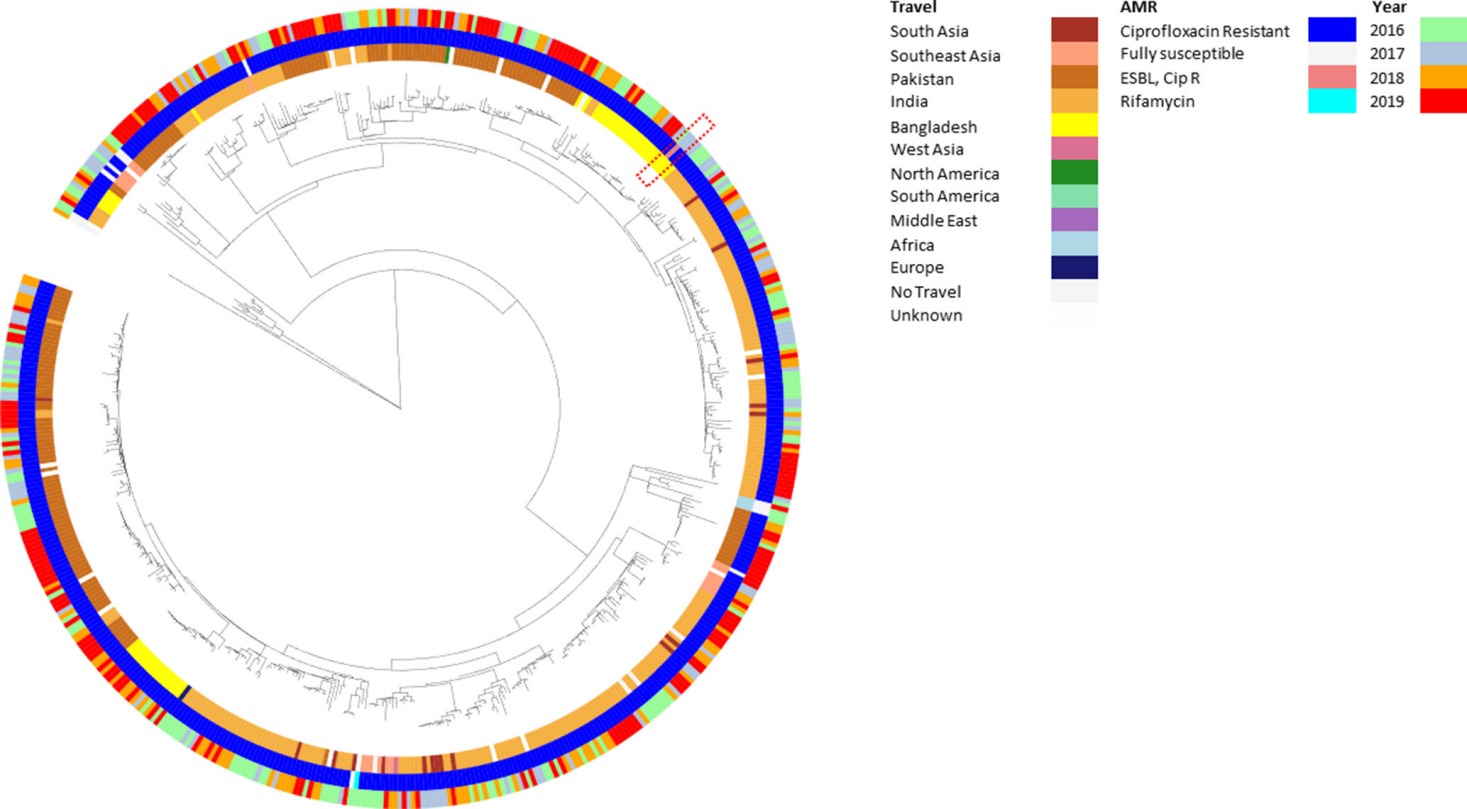
Neighbour-joining phylogeny of *S*. Paratyphi A in association with year of receipt, AMR and travel. Phylogenetic tree of cgMLST analysis of *S*. Paratyphi A strains generated using the neighbour-joining method and mapped against three categories: inner ring, travel; middle ring, AMR (Cip R, ciprofloxacin-resistant strains; ESBL, extended-spectrum β-lactamase producing strains); outer ring, year. The phylogeny shows multiple clusters of ciprofloxacin-resistant strains mainly associated with travel to India, Pakistan and Bangladesh. One strain falling into a cluster associated with travel to Bangladesh was seen in 2017 but has not been imported since (red dashed line box).

*S*. Paratyphi A cases fell in 2017 and then increased in 2018 and 2019, and were predominantly ciprofloxacin resistant (*n*=522/529 cases, 98.7 %) (Fig. 6). Resistance was most commonly caused by double mutations in the QRDR of *gyrA* and *parC*, specifically *gyrA*[83:S-F];*parC*[57:T-S] (*n*=393, 74.3 %) or *gyrA*[83:S-Y];*parC*[57:T-S] (*n*=120, 22.7%). Triple mutations in the QRDR were also found with the most common being *gyrA*[83:S-F;87:D-G];*parC*[57:T-S] (*n*=5, 0.9 %) (Tables 3 and S1). There were multiple clusters distributed throughout the population structure associated with patients reporting travel to India (*n*=258/529, 48.8 %), followed by travel to Pakistan (*n*=173/529, 32.7 %) and Bangladesh (*n*=49/529, 9.3 %) (Figs 7 and S2a, b, Table S3).

**Fig. 8.**
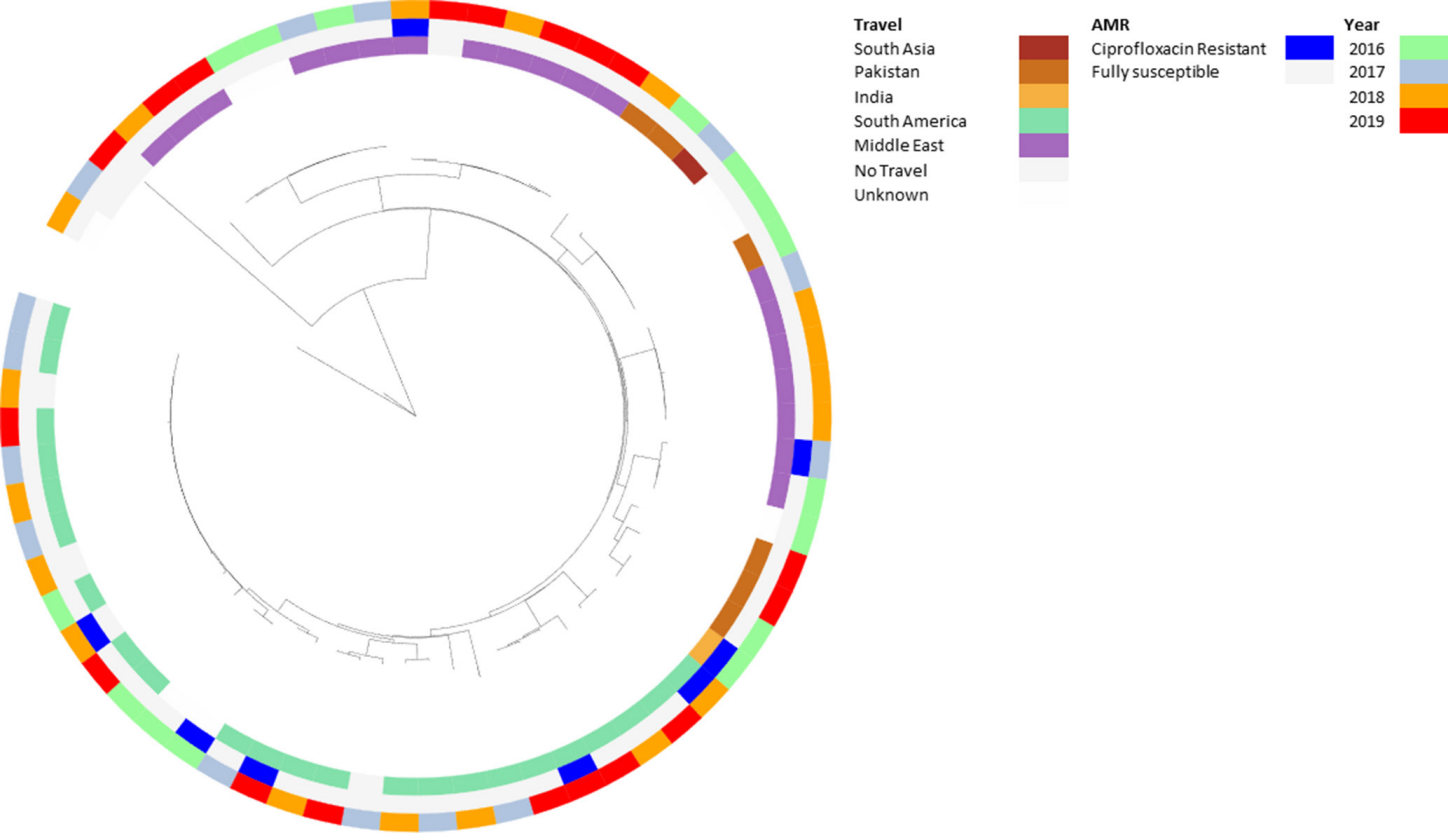
Neighbour-joining phylogeny of *S*. Paratyphi B in association with year of receipt, AMR and travel. Phylogenetic tree of cgMLST analysis of *S*. Paratyphi B strains generated using the neighbour-joining method and mapped against three categories: inner ring, travel; middle ring, AMR; outer ring, year. The phylogeny shows one main clade associated with travel to South America and one main clade associated with travel to the Middle East containing two sub-clusters mainly associated with travel to Pakistan. Resistance to ciprofloxacin occurs sporadically throughout the population.

Of the 67 isolates from cases of *S*. Paratyphi B, 8 (11.9 %) isolates were resistant to ciprofloxacin (MIC ≥0.125 mg l^−1^) and either had a single mutation in *gyrA*[83:S-F] (*n*=6, 9 %), a single mutation in *gyrA*[87:D-N] (*n*=1, 1.5 %) or had the *qnrB19* Plasmid-Mediated Quinolone Resistance (PMQR) determinant (*n*=1, 1.5 %) (Table S1). Ciprofloxacin resistance was sporadically distributed throughout the population structure and associated with multiple destinations of travel. Though numbers of *S*. Paratyphi B were small, there were multiple clonal groups that could be distinguished with travel to specific countries continuing for several years (Figs 8 and S3a, b, Table S3). There were no genes predicted to confer resistance to ciprofloxacin resistance in *S.* Paratyphi C (Table S1).

One *S*. Paratyphi A isolate had a single mutation *rpoB*[516:D-G] encoding rifamycin resistance and was phenotypically resistant [MIC >32 mg l^−1^] that was not previously detected in the Day *et al.* (2018) study [9]. No genotypic or phenotypic resistance was detected in *S*. Paratyphi A, B or C to macrolides, fosfomycin or colistin (Tables 3 and S3).

## Discussion

This study has utilized genomic data routinely generated at PHE to continue to validate phenotypic predictions and better understand the trends, burden, AMR and phylogenomics of *S*. Typhi, *S*. Paratyphi A and *S*. Paratyphi B isolated from returning travellers in England. The use of genomics to detect AMR determinants and predict phenotypic resistance has been well described [9, 20, 34], and this study confirms that use of genome data is a robust and accurate approach with 99.99 % concordance between genotypic and phenotypic resistance for the typhoidal salmonellae. Surveillance of genome-derived AMR profiles enables the real-time monitoring of the emergence and spread of AMR determinants in all isolates referred without added cost.

Phenotypic testing still plays a vital role, as we continue to see instances where AMR genes expected to confer resistance to a specific antimicrobial class may be present in isolates that do not exhibit phenotypic resistance, often due to mutations or indels rendering the gene non-functional (Tables 1 and S1). Since the last reported study between 2014 and 2016 [9] where only one PMQR determinant (*qnrB19*) was found in a single *S*. Typhi isolate, there has been acquisition and increase in multiple PMQR determinants across the population (*qnrS-1*, *n*=56; *qnrB-*7, *n*=1; q*nrB*-19, *n*=1; Table S1). Though the value is high for using WGS for screening large amounts of data, it is still essential to maintain phenotypic testing for different pathogens, not only to monitor for emerging novel resistance mechanisms, but also to facilitate accurate interpretation of genome-derived AMR profiles.

The increase of *S*. Typhi cases corresponds with an increase in the proportion of strains of *S*. Typhi exhibiting resistance to the majority of antibiotic classes (Table 1, Fig. 3). Of most concern was the increase in resistance to ciprofloxacin and the third-generation cephalosporins (Fig. 4), key components of treatment regimens for enteric fever. The increase of MDR strains, first reported in the 1990s [16, 35], as well the emergence of XDR strains, has been documented in other studies reporting these increases in relation to travel to South Asia [8, 36]. In this study, the increase in incidence of MDR and XDR *S*. Typhi strains in 2019 was associated with the HC_1452 (H58) clone in association with travel to Pakistan (Fig. 2), as previously described by Klemm *et al*. [8], and demonstrates the value of monitoring AMR in returning travellers. Although *S*. Typhi is considered monomorphic and there are clear associations with clades to geographical travel and acquisition of resistance, the resistance mechanisms observed included a combination of plasmid acquisition, point mutations and chromosomal integration [37] (Figs 5 and S1a–c, Table 1).

For *S*. Paratyphi A, the most notable increase of cases was from 2018 to 2019, with genomic analysis showing ciprofloxacin-resistant clones from India and Pakistan being the most commonly imported strains (Figs 6, 7 and S2a, b, Table S3). In line with data from previous studies [36], the *S*. Paratyphi A population has consistently remained ciprofloxacin resistant (*n*=522/535, 97.6 %) (Table 3), exhibiting the same double mutations in *gyrA* and *parC* genes [9] throughout the population structure (Figs 7 and S2a, b). This may be due to the QRDR being prone to the same mutation in *S*. Paratyphi A under selective pressure. Though MDR strains of *S*. Paratyphi A in Southeast Asia have been reported [36], there was only one ESBL-producing isolate in this study from a case reporting recent travel to Bangladesh [6]. This contrasts the impact of imported clones of *S*. Typhi with multidrug resistance not only in terms of resistance but also with the number of cases, despite the similar travel destinations with these two serovars. This may be due to the success of the clonal expansion of the HC_1452 (4.3.1/H58) *S*. Typhi strain which has now spread across the globe [12], if this occurs with the MDR *S*. Paratyphi A, then we are likely to see an increase of imported cases.

*S*. Paratyphi B case numbers remained relatively stable and isolates have remained susceptible to antibiotics since 2015. *S*. Paratyphi B population structure also showed HC5 clonal groups associated with travel, but to different destinations than *S*. Typhi and *S*. Paratyphi A, as most cases were associated with travel to the Middle East and South America (Table S3, Figs 8 and S3a, b). Though the different destinations of travel may explain the smaller number of *S*. Paratyphi B cases in England, there were two clusters associated with travel to Pakistan, so why are the number of cases relatively low? One reason could be that although *S*. Paratyphi B may be endemic in Pakistan, it is not as prevalent as *S*. Typhi and *S*. Paratyphi A and, therefore, less cases are imported into England. *S*. Paratyphi B are not human host restricted [38] and an alternative theory for fewer reported cases is that symptoms associated with *S*. Paratyphi B infection are less severe (hence, the lowest invasive index [39]) and people are less likely to seek health care.

This study is a focus on the trends of enteric fever in England, and although these data can be used as a surrogate sentinel AMR surveillance to assess emerging trends of resistance in other countries [10], real-time comparison of global data would continue to validate this approach and detect new clones. HierCC at the HC5 level [13] has been shown in this study to be a useful tool and typing scheme in assessing clonal groups across all enteric fever pathogens and linking population structure to case demographics [12]. EnteroBase can also be used to search for other related HC5 strains, enabling the user to put their data in the global context [13, 24]. Another global platform that can be used is the NCBI Pathogen Detection platform (www.ncbi.nlm.nih.gov/pathogens; www.ncbi.nlm.nih.gov/pathogens/antimicrobial-resistance/AMRFinder/) where you can look for related strains and detect antimicrobial markers.

This review of genomic-resistance trends in enteric fever provides a robust evidence base for reviewing and updating clinical guidelines, particularly where there has been travel to specific regions. The analysis described here has highlighted the changing trends of resistance in *S*. Typhi and further analysis has been undertaken using prescriptive statistics (T. Herdman, B Karo, J Dave, P Katwa, J Freedman, et al., unpublished results) to guide and update clinical guidance for treatment of enteric fever in England and Wales. The World Health Organization (WHO) currently recommends chloramphenicol, ampicillin and cotrimoxazole (trimethoprim/sulphamethoxazole), fluoroquinolones, third-generation cephalosporines (ceftriaxone, cefixime) and azithromycin for the treatment of enteric fever [36, 40]. This study has highlighted the importation of XDR *S*. Typhi from Pakistan [5, 37] requiring treatment options such as the use of azithromycin as first-line treatment until results of phenotypic susceptibility testing are available. The recommendation of empirical treatment with the use of third-generation cephalosporins with *S*. Typhi would be recommended where cases are not imported from XDR endemic areas (T. Herdman, B Karo, J Dave, P Katwa, J Freedman, et al., unpublished results). Carbapenems would, henceforth, be the best option for empirical treatment of enteric fever for cases imported from Pakistan, until the antimicrobial-susceptibility profile is determined. Azithromycin continues to be a reliable treatment option for treatment of uncomplicated enteric fever, there was no reported resistance in this study, although other studies from endemic areas have reported higher resistance rates from in India (1–34 %) and Pakistan (85 %) [36, 41–43]. Fortunately, the carriage status of infection did not appear to have an impact on acquiring additional resistance mechanisms. Though only a handful of cases were associated with chronic carriage, up to 20 % of typhoidal *Salmonella* caused convalescent carriage and this is important to consider when undertaking public-health action (Table 2).

### Conclusions

This study provides further evidence that genome-derived AMR profiling is a robust approach for rapidly predicting phenotypic resistance and enables routine prospective surveillance in countries who have the resources to undertake this methodology. Genomic surveillance of typhoidal salmonellae strains continues to be a useful tool, and HierCC can be used to define clones and link expanding resistant clones in association with travel to endemic countries. Rapid detection of emerging mechanisms of resistance, like XDR strains from Pakistan, is crucial for effective management of imported infections and plays an important role in informing treatment guidelines. Genomic surveillance also continues to play an important role in other prevention strategies, like development of effective vaccines and other public-health measures.

## Supplementary Data

Supplementary material 1

Supplementary material 2
