# Supplementary material for: Phylogenomics and antimicrobial resistance of Salmonella Typhi and Paratyphi A, B and C in England, 2016–2019
Source: Microb Genom. 2021 Aug 9;7(8):000633. doi: 10.1099/mgen.0.000633 (PMC8549371; doi:10.1099/mgen.0.000633)
Supplement: Supplementary material 1 [file mgen-7-0633-s001.pdf]

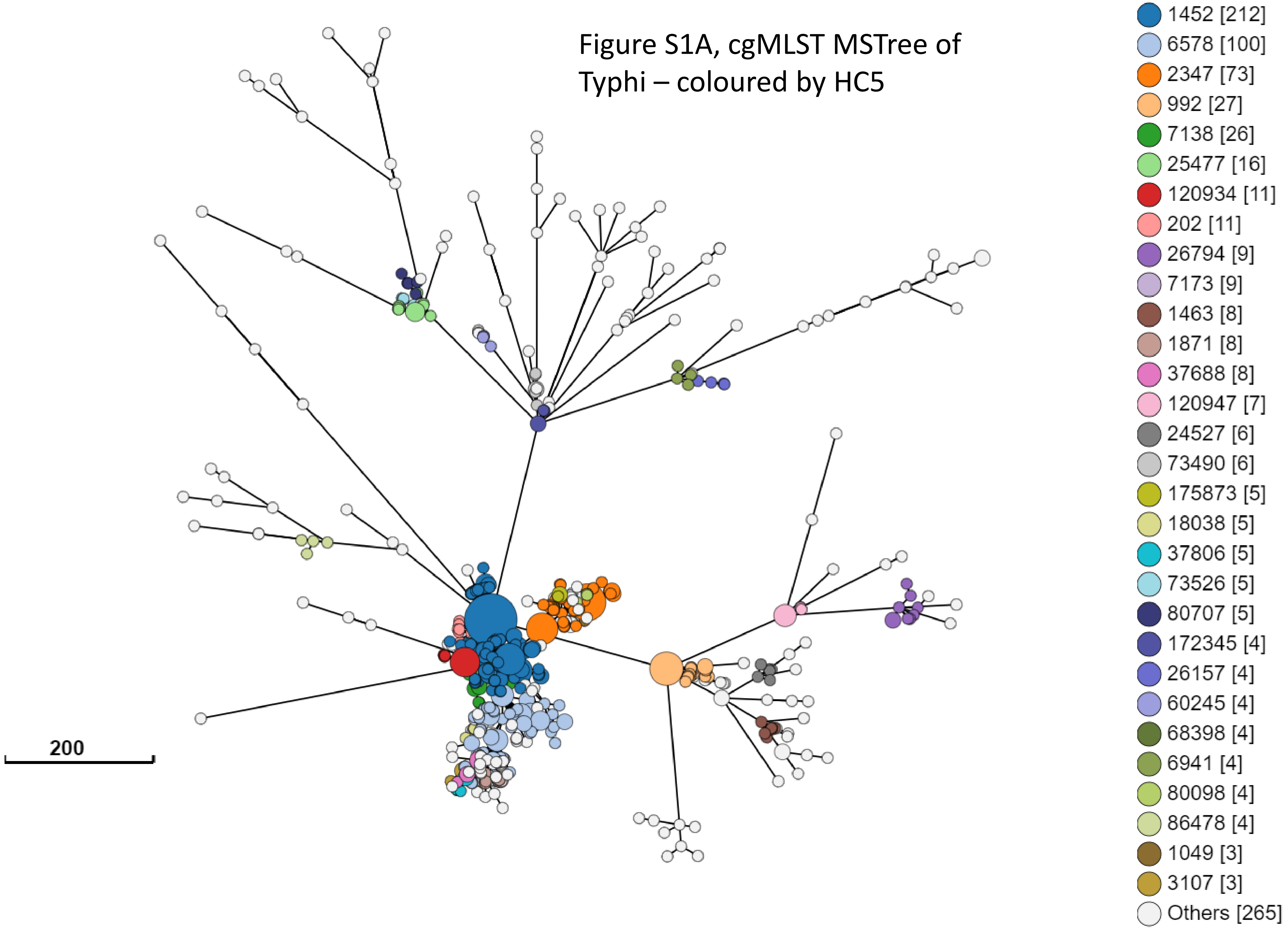

Figure S1A, cgMLST MSTree of Typhi – coloured by HC5
200
1452 [212]
6578 [100]
2347 [73]
992 [27]
7138 [26]
25477 [16]
120934 [11]
202 [11]
26794 [9]
7173 [9]
1463 [8]
1871 [8]
37688 [8]
120947 [7]
24527 [6]
73490 [6]
175873 [5]
18038 [5]
37806 [5]
73526 [5]
80707 [5]
172345 [4]
26157 [4]
60245 [4]
68398 [4]
6941 [4]
80098 [4]
86478 [4]
1049 [3]
3107 [3]
Others [265]

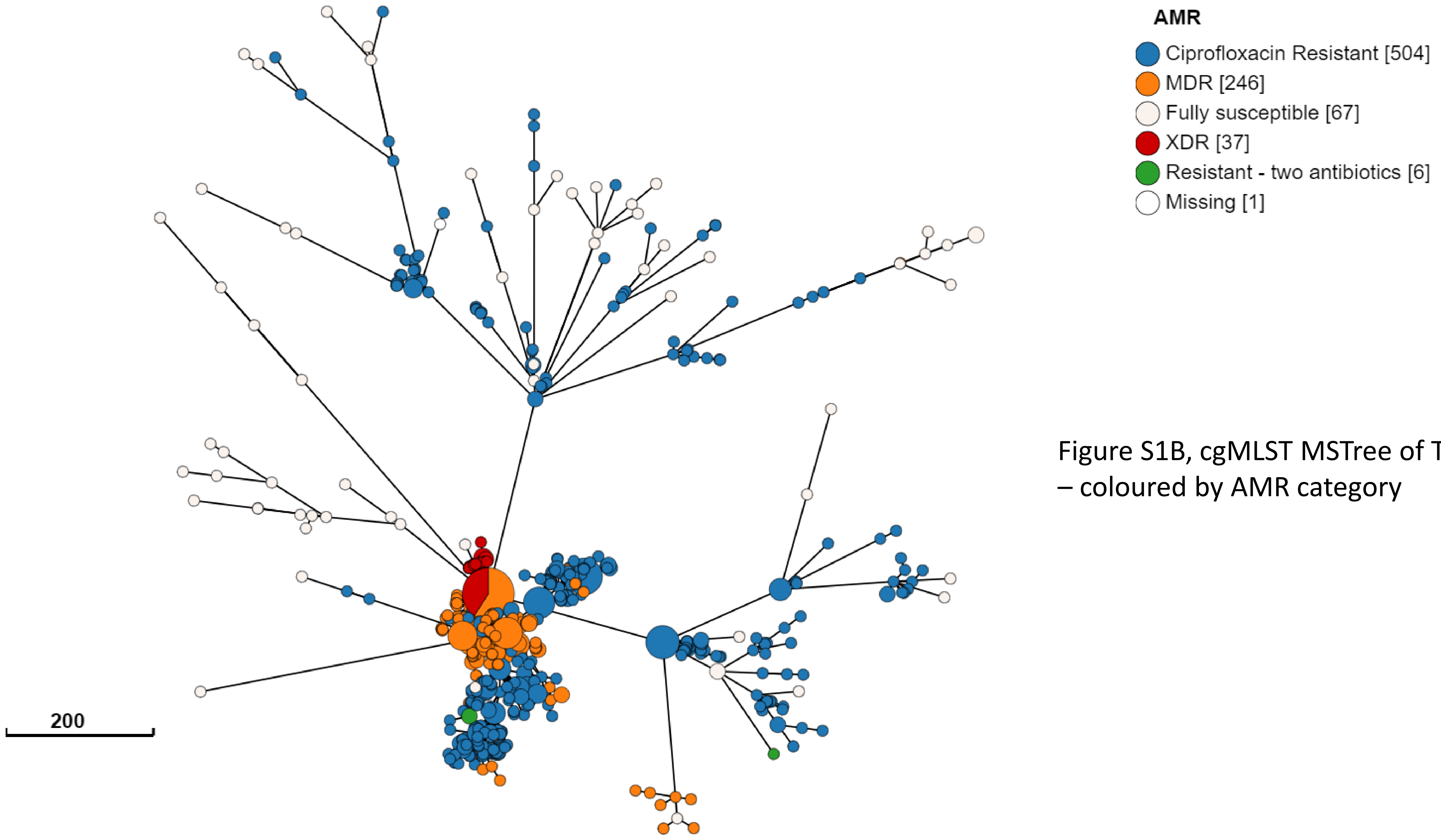

Figure S1B, cgMLST MSTree of Typhi – coloured by AMR category

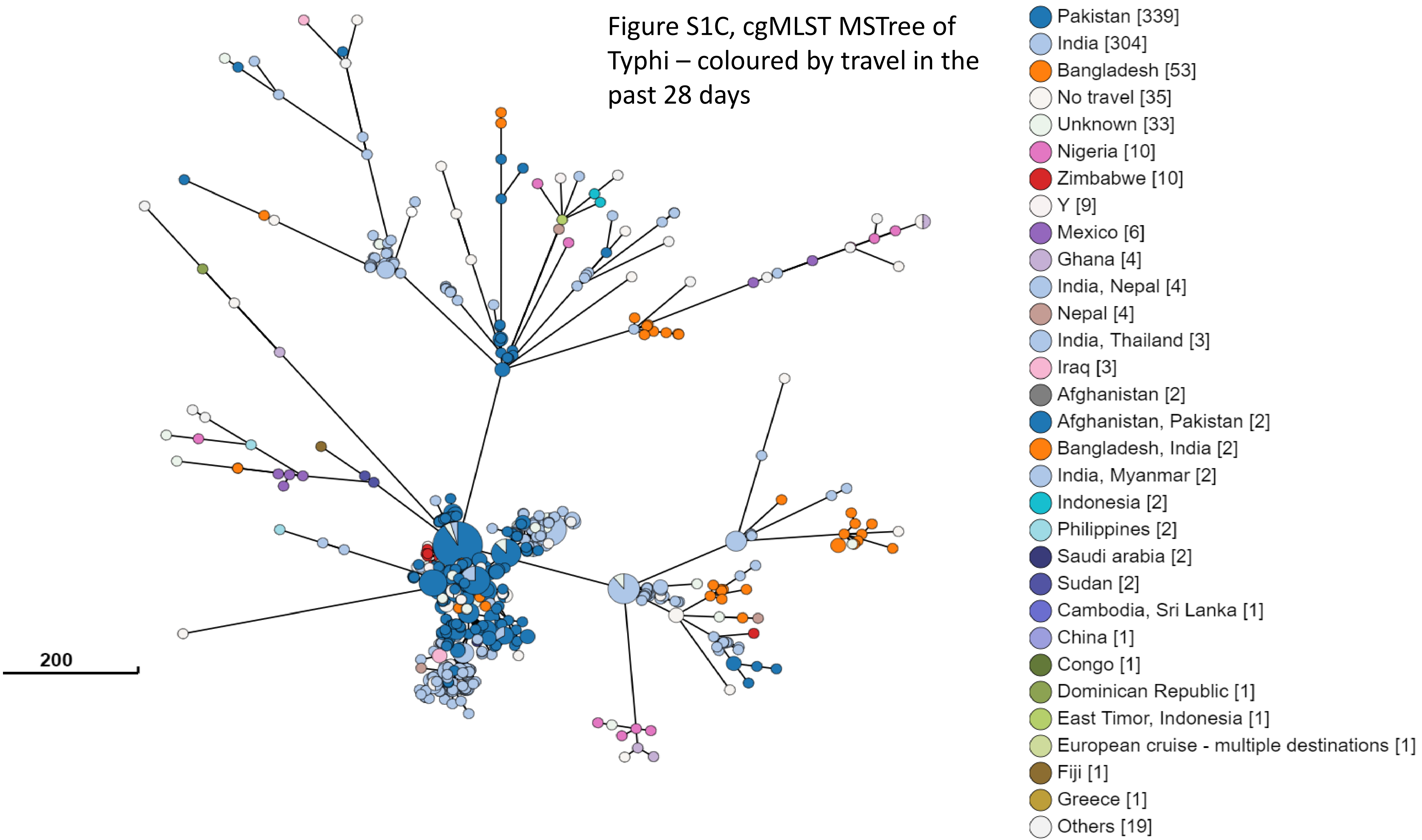
Figure S1C, cgMLST MSTree of Typhi – coloured by travel in the past 28 days
200
Pakistan [339]
India [304]
Bangladesh [53]
No travel [35]
Unknown [33]
Nigeria [10]
Zimbabwe [10]
Y [9]
Mexico [6]
Ghana [4]
India, Nepal [4]
Nepal [4]
India, Thailand [3]
Iraq [3]
Afghanistan [2]
Afghanistan, Pakistan [2]
Bangladesh, India [2]
India, Myanmar [2]
Indonesia [2]
Philippines [2]
Saudi arabia [2]
Sudan [2]
Cambodia, Sri Lanka [1]
China [1]
Congo [1]
Dominican Republic [1]
East Timor, Indonesia [1]
European cruise - multiple destinations [1]
Fiji [1]
Greece [1]
Others [19]

Figure S2A cgMLST MSTree of Paratyphi A – coloured by cgMLST

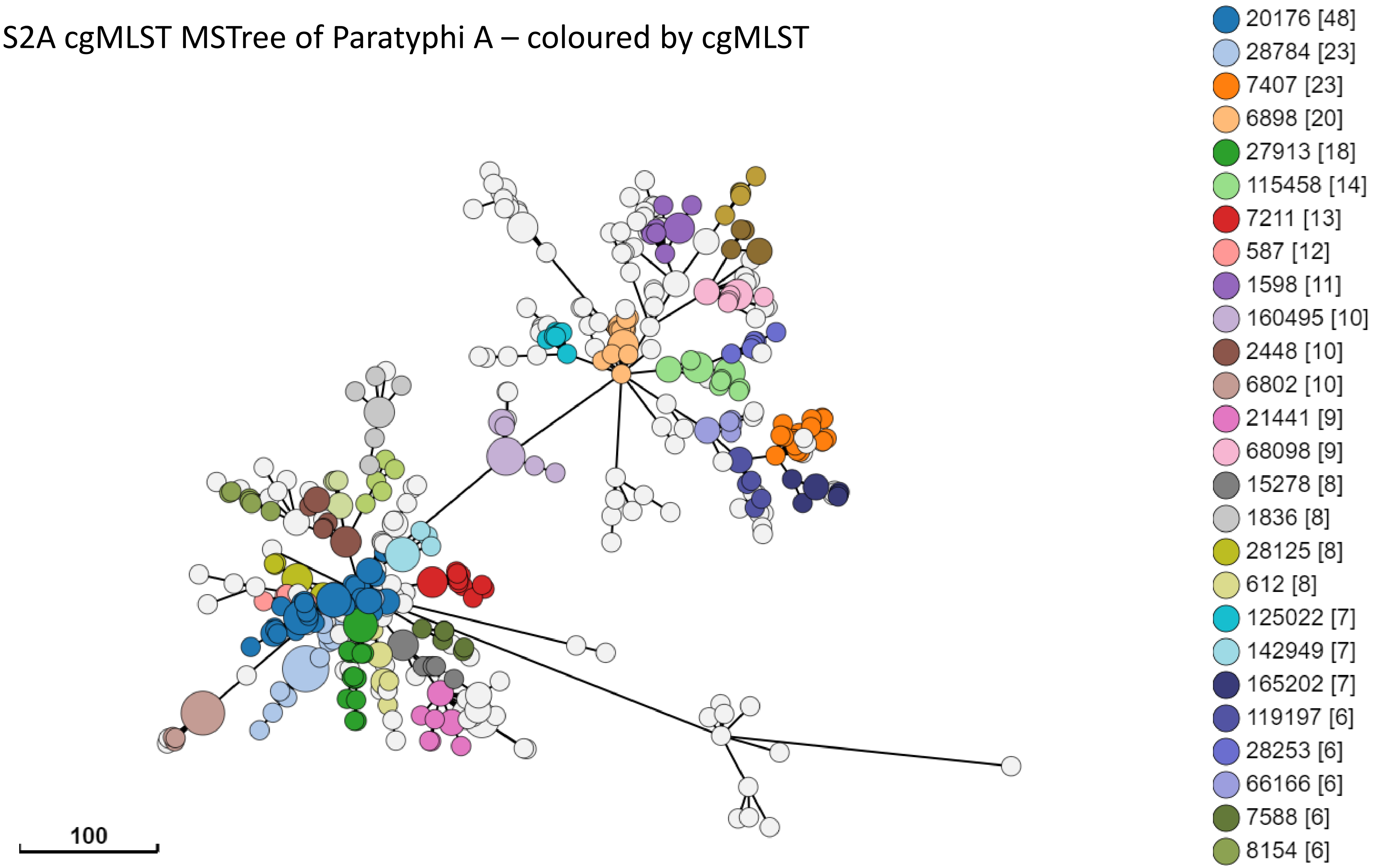

Figure S2B, cgMLST MSTree of Paratyphi A – coloured by travel in the past 28 days

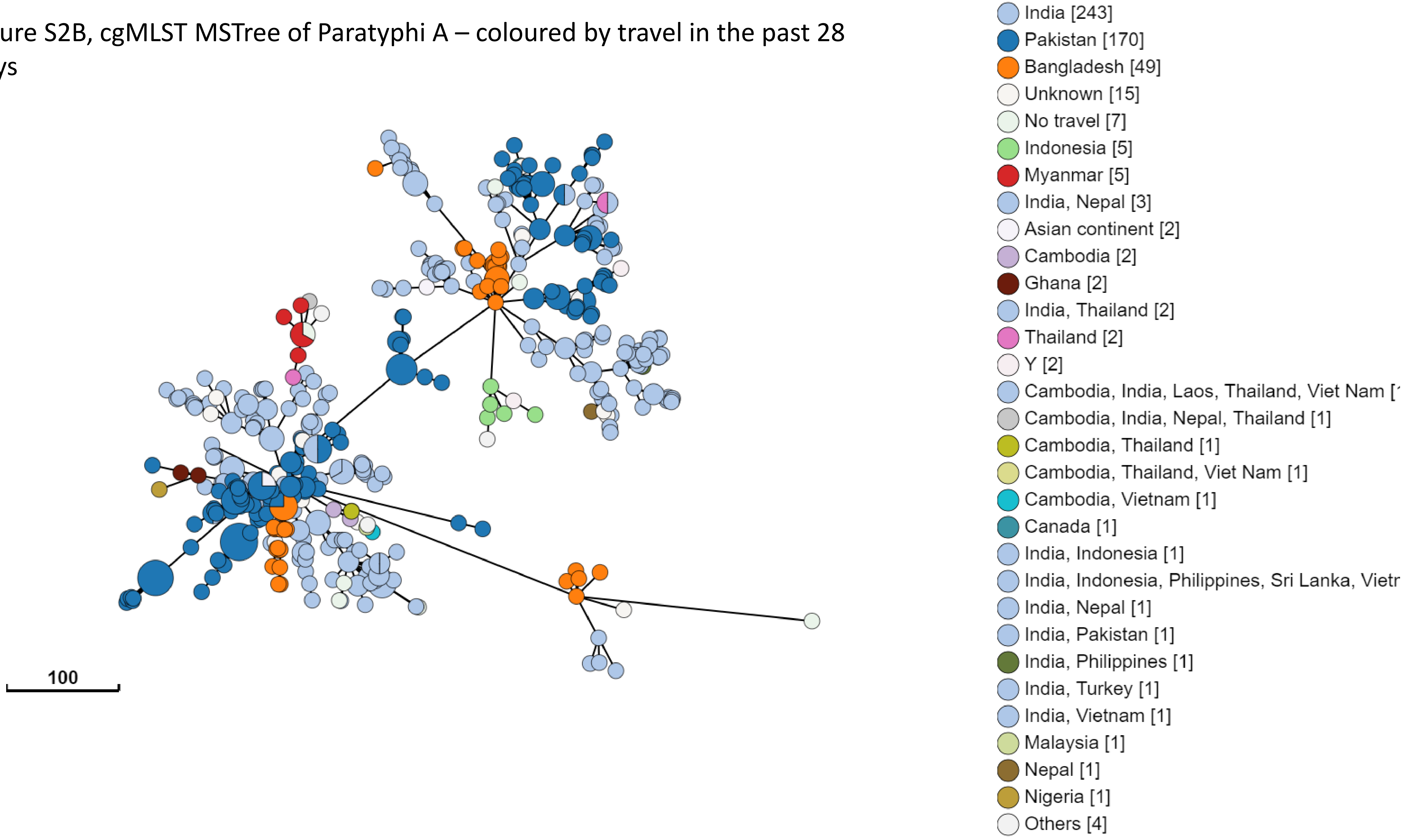

Figure S3A, cgMLST MSTree of Paratyphi B – coloured by cgMLST HC5

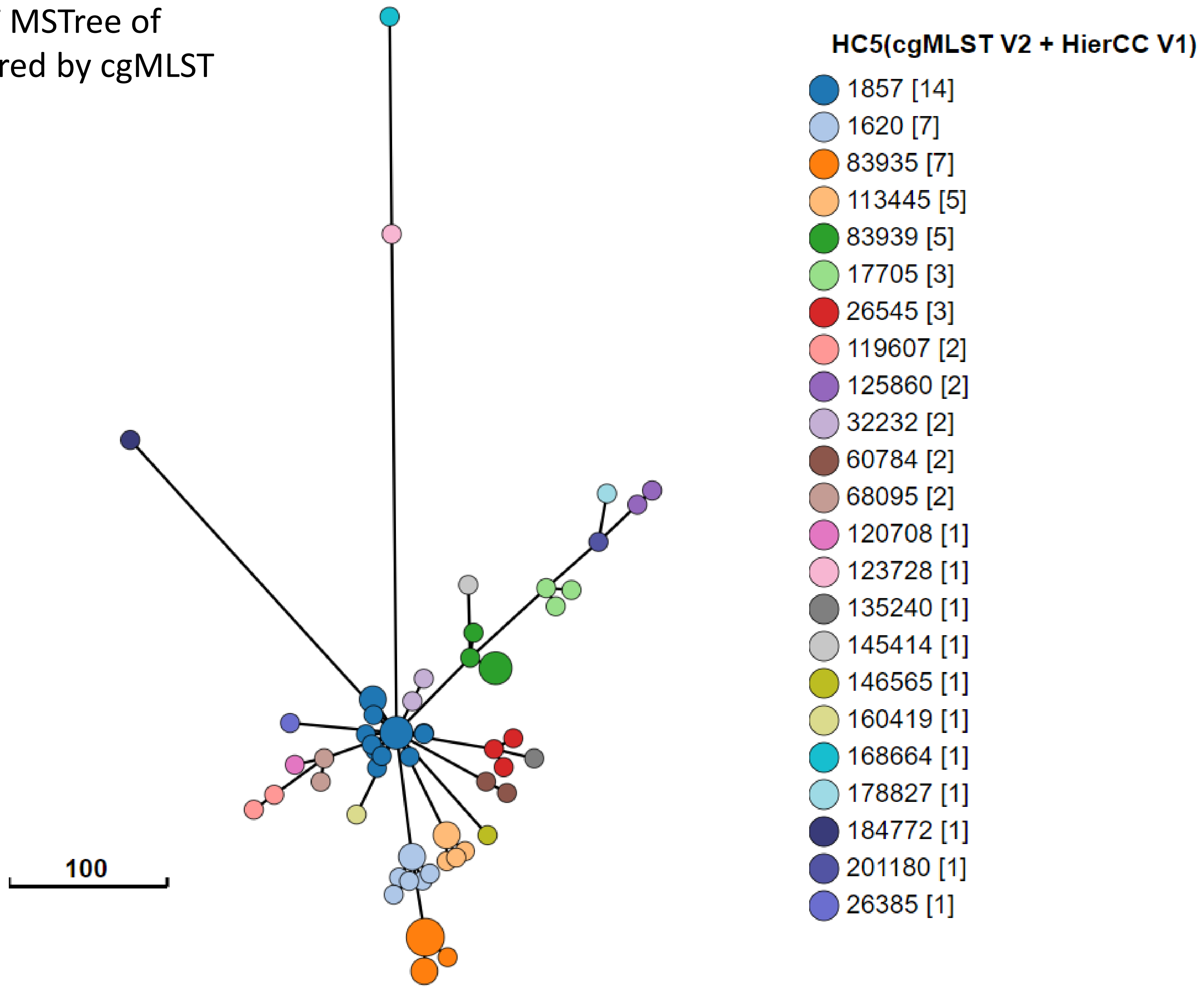

Figure S3A, cgMLST MSTree of Paratyphi B – coloured by travel in the past 28 days

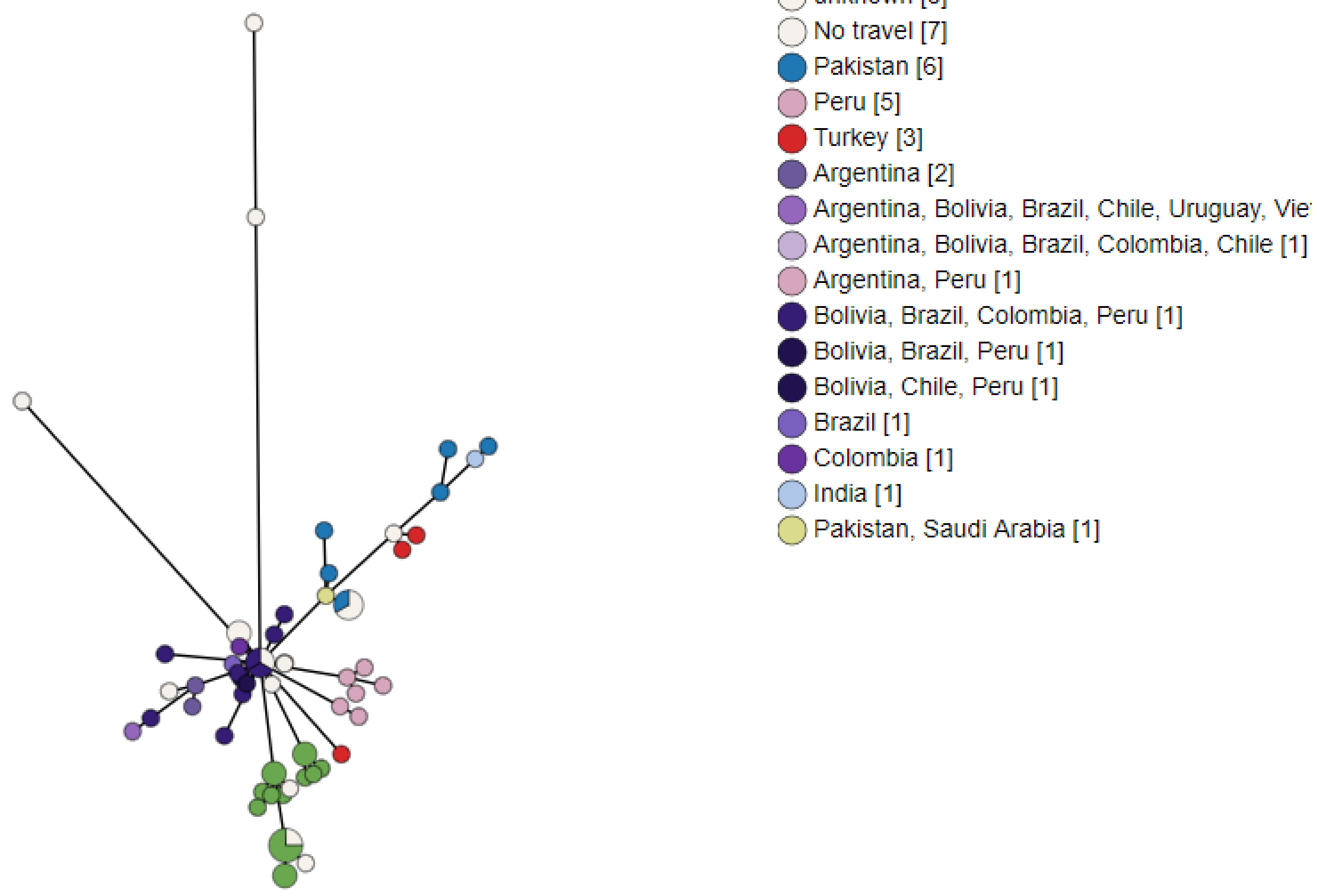

Legend: Hierarchical Clustering (HierCC) of enteric fever cases in England between 2016-2019. GrapeTree depiction of MSTree tree V2 of cgMLST allelic distances between genomic entries, coloured by different categories: *S.* Typhi HierCC number at the HC5 allelic level (S1A), *S.* Typhi and AMR category (S1B), *S.* Typhi and travel in the past 28 days (S1C), *S.* Paratyphi A and HierCC number at the HC5 allelic level (S2A), *S.* Paratyphi A and travel in the past 28 days (S2B),), *S.* Paratyphi B and HierCC number at the HC5 allelic level (S3A), *S.* Paratyphi B and travel in the past 28 days (S3B).
